# A new era in computational pathology: A survey on foundation and vision-language models

**DOI:** 10.1016/j.jpi.2026.100714

**Published:** 2026-09-08

**Authors:** Dibaloke Chanda, Milan Aryal, Masoud Ganji, Anil Parwani, Nasim Yahya Soltani

**Affiliations:** a Department of Computer Science, Marquette University, Milwaukee, WI 53233, USA; b North Shore Pathologists, S.C., Milwaukee, WI 53211, USA; c Department of Pathology, The Ohio State University, Columbus, OH 43210, USA

**Keywords:** Computational pathology, Foundation models, Multi-modal, Vision-language models

## Abstract

Recent advances in deep learning have completely transformed the domain of computational pathology (CPath). More specifically, it has altered the diagnostic workflow of pathologists by integrating foundation models (FMs) and vision-language models (VLMs) in their assessment and decision-making process. The limitations of existing deep learning approaches in CPath can be overcome by FMs through learning a representation space that can be adapted to a wide variety of downstream tasks without explicit supervision. Deploying VLMs allow pathology reports written in natural language be used as rich semantic information sources to improve existing models as well as generate predictions in natural language form. In this survey, a holistic and systematic overview of recent innovations in FMs and VLMs in CPath is presented. Furthermore, the tools, datasets, and training schemes for these models are summarized in addition to categorizing them into distinct groups. This extensive survey highlights the current trends in CPath and its possible revolution through the use of FMs and VLMs in the future.

## Introduction

In recent years, there has been a surge of artificial intelligence (AI)-based approaches^1^, ^2^, ^3^, ^4^, ^5^, ^6^, ^7^, ^8^, ^9^, ^10^, ^11^, ^12^, ^13^, ^14^, ^15^, ^16^, ^17^, ^18^, ^19^ in computational pathology (CPath) owing to wide adoption of digital slide scanners. In fact, large-scale curation and annotation^20^, ^21^ of whole-slide images (WSIs) have ensured adequate data for training of these AI-based models. The goal of generating such AI-based models is to automate and expedite the diagnosis and prognosis process of CPath. Traditional diagnosis process in CPath is time-consuming and requires experts with extensive domain knowledge. In addition, the wide range of cancer types and grades as well as the heterogeneity among diagnosis/prognosis tasks, makes it challenging to come up with a unified general approach.

Several research studies have addressed the issue with a unified approach and among the proposed methods, foundation models (FMs) have gained a lot of attention in recent years.^22^, ^23^, ^24^, ^25^, ^26^, ^27^, ^28^, ^29^, ^30^, ^31^, ^32^, ^33^, ^34^, ^35^, ^36^, ^37^, ^38^, ^39^, ^40^, ^41^, ^42^ More specifically, FMs leverage self-supervised learning (SSL)^43^ schemes to learn a rich representation in a task-agnostic manner. Owing to self-supervised pre-training (SSPT), FMs do not require large-scale annotated data, which are hard to come by in CPath. Furthermore, these models can be trained with a diverse selection of datasets containing tissue samples from different organs and associated with different cancer types, as well as scanner types. Certain research works focus on making sure this diversity is present in the pre-training dataset.^26^, ^29^ As a result, the resultant pre-trained model can easily be utilized in a wide range of downstream tasks while maintaining robustness to extreme variation in tissue samples.

The impact of FMs in CPath can be amplified by integrating the power of vision-language models (VLMs).^44^ More specifically, VLMs have surged in popularity after the introduction of contrastive language-image pre-training (CLIP) model by OpenAI.^45^ Pathology reports, books, educational videos, etc. are rich sources of semantic information that can be utilized by VLMs to enhance model capabilities beyond what is achievable with vision-only models. Because VLMs learn from both images and text together, they can pick up on important clinical details such as diagnostic descriptions, tissue-level terminology, and prognostic information that vision-only models have no way of accessing. This joint learning enables VLMs to build richer representations that connect visual patterns in tissue images with their corresponding clinical meaning, leading to a more holistic understanding of the data. Although pure FMs may still outperform VLMs on certain downstream tasks due to their focused training on visual features, VLMs offer a unique advantage by supporting tasks that would otherwise not be possible, such as classifying images without task-specific training, retrieving images using natural language queries, and producing more interpretable results. In particular, the ability of VLMs to operate in an open-vocabulary setting makes them highly adaptable to new and unseen diagnostic categories without the need for additional labeled data. When deployed in conjunction with FMs, they can perform as AI-based pathologists capable of performing a vast array of tasks as evidenced by recent literature.^37^ Even though CPath is a specialized area of study in the medical field, there is a large increase in the number of publications focusing on FMs and VLMs in pathology as shown in Fig. 1 demonstrating a dominant future direction of this field. The reason for surveying FMs and VLMs together is due to commonality in several aspects including large-scale pre-training, complementary modality (i.e., image and text), similar architectural component, impact on clinical workflows, which includes helping pathologists through image analysis, report generation, and decision support, overlapping challenges, which includes domain adaptation, robustness, and interpretability, etc. Furthermore, recent trend show the models that are most prominent and ready for practical use in clinical settings are vision-language foundation models (VLFMs), which is the overlap between FMs and VLMs. Recently emerging tools like PathChat 2^46^ and Judith^47^ by Modella AI provide further evidence into how the future of CPath will look like with the combination of FMs and VLMs.

**Fig. 1:**
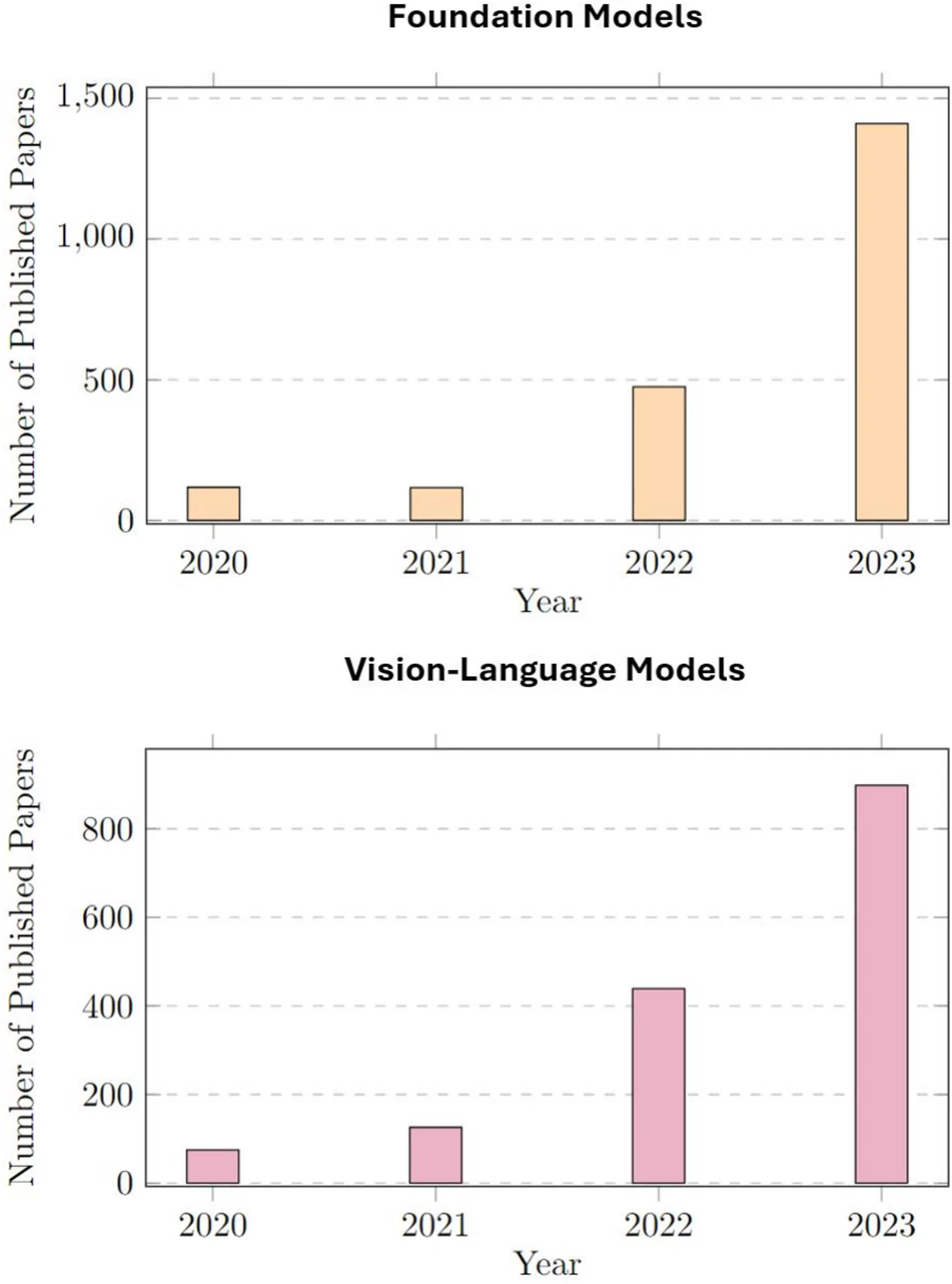
Number of publications for FMs and VLMs in pathology (from Google Scholar). The search keywords include “vision-language” + “pathology” for VLMs statistics and “foundation models” + “pathology” for FMs statistics.

To appreciate the full impact of FMs and VLMs in CPath, the major challenges in CPath are outlined in Fig. 2. In addition, the way FMs and VLMs address these challenges is also mentioned.

**Fig. 2:**
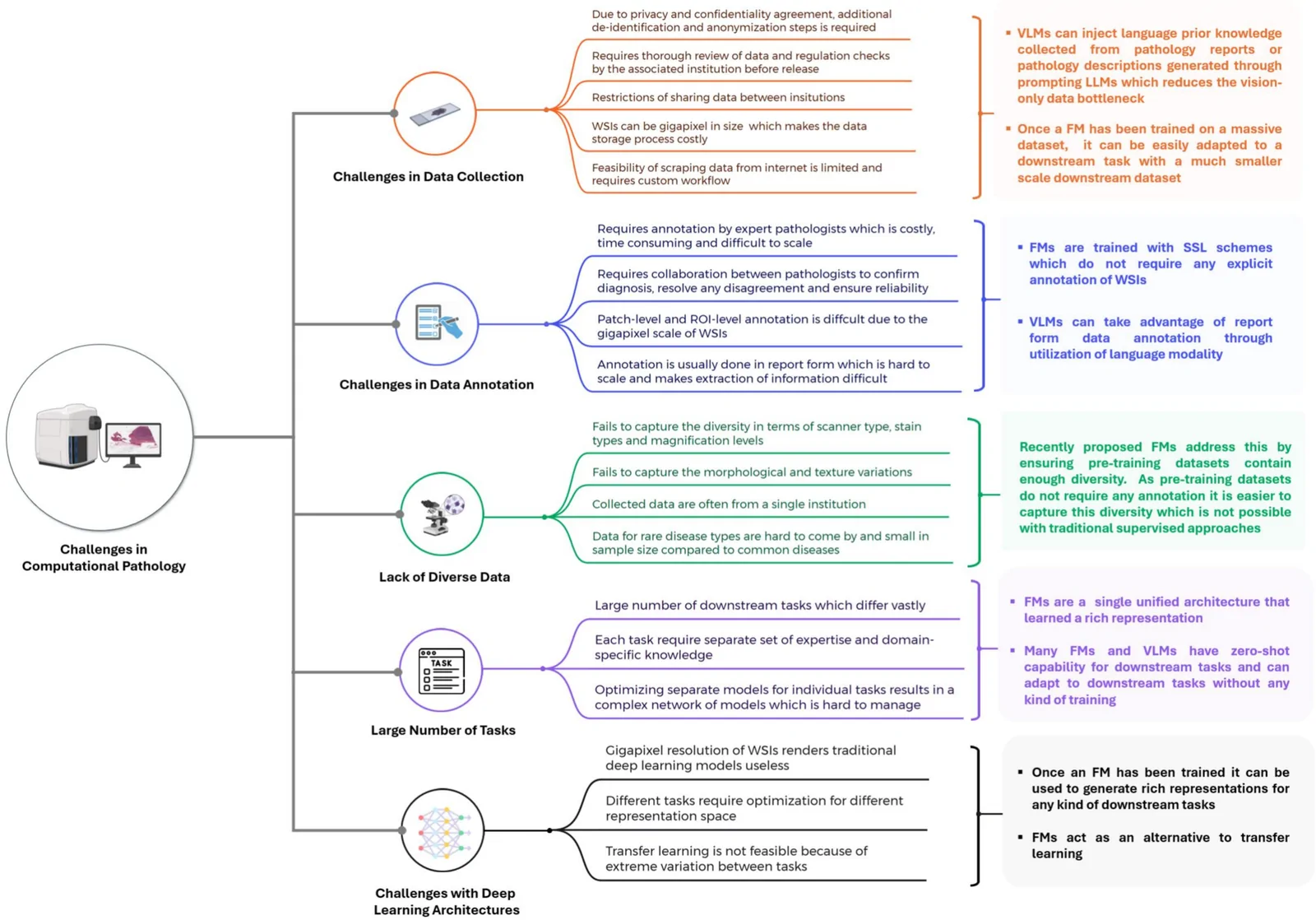
Outline of major challenges in CPath. Several causes and consequences for each challenge are outlined, in addition to how FMs and VLMs address these challenges.

### Scope of the review

In this review, the main emphasis is put on the application of FMs and VLMs in CPath, especially the details of their architectures and training schemes. Note that these two categories are not mutually exclusive, meaning that some research articles belong to both categories, which are VLFMs. In addition, details of multi-modal datasets are summarized with a focus on vision and language as the modalities. Several self-imposed rules and restrictions were used as guidelines throughout the review to ensure the scope of the article is maintained.

1. First, articles that focus solely on pathology are included. So, and articles with a focus on other areas of the biomedical domain are excluded. As an example, articles like BiomedCLIP^48^ with pathology as a subsection of the research are not included in this review article.
2. Second, only VLMs are included and articles that use other modalities along with vision are excluded. As an example, along with vision, transcriptomics^49^ can be used to address pathology tasks. However, such articles are excluded to maintain the scope.
3. Both peer-reviewed articles and pre-print articles are included in the survey. Among the peer-reviewed articles, a significant number of articles are published in top-tier journals and conferences as shown in Table 1.

**Table 1:** Number of surveyed articles published in top journals and conferences within 2021–2024.

Journal/Conference venue	Counts
Nature/Nature Medicine	
CVPR	
NeurIPS	
ECCV/WACV/ICCV	
MICCAI	
AAAI	
Elsevier/Springer

### Contribution and organization

In the past few years, quite a few review articles^1^, ^2^, ^3^, ^4^, ^5^, ^6^, ^7^, ^8^, ^9^, ^10^, ^11^, ^12^, ^13^, ^14^, ^15^, ^16^, ^17^, ^18^, ^19^ have been published focusing on CPath. Most of these articles provide a review of the application of deep learning as a whole rather than focusing on a specific subtopic. There are handful of articles that focus on specific architecture like MIL,^18^ graph-based models,^17^, ^50^ transformer-based models,^10^, ^13^ LLMs,^19^ etc. The contributions of this review article are summarized below:

1. To the best of our knowledge, this is the first review article to summarize recent advances (most articles from 2023 to 2024) in FMs and VLMs (Foundation model and Vision-language models). The closest peer-reviewed survey article^8^ mostly provides a high-level overview without going into the details of FMs.
2. An exhaustive list of multi-modal datasets (Section 2) in CPath that are being used or can be used in vision-language research are outlined.
3. Given the diverse datasets and architectures, descriptions for individual research are provided in tabular format (Table 2, Table 4, Table 5, Table 9), so it is easier for readers to follow.

**Table 2:** Summary of multi-modal datasets in pathology.

Dataset	Type of data	Size	Image and text source	Method of generation/Procurement	Other utilized dataset/Models for generation/Procurement	Dataset used by	Variation/Subset/Extension	Availability
WSI-VQA^51^	WSI and Q/A pairs	977 WSIs 8672 question–answer pairs (slide-level) with average 8.9 Q/A pairs per WSI	WSI and pathology report from TCGA-BRCA	Prompting LLMs and template matching heuristics	**Model/Framework:** GPT-4^52^	Wsi2Text Transformer (W2T)^51^	4535 close-ended VQA subset and 4137 open-ended VQA subset	√
PathGen-1.6 M^53^	Image-caption pairs	1.6 million	WSI and pathology report from TCGA	Multi-agent collaboration and caption generation with large multi-modal models (LMMs)	**Dataset:** PathCap,^40^ Quilt-1M,^54^ OpenPath^42^ **Model/E**V**amework:** LLaVA-v1.5 ^55^, Vicuna^56^ OpenCLIP,^57^ GPT-4^52^	PathGen-CLIP,^53^ PathGen-LLaVA^53^	200 K instruction-tuning data (Extension)	√
Quilt-Instruct^58^	Instruction-tuning question–answer pairs	107,131 histopathology-specific Q/A pairs	Over 1000 h of 4149 educational histopathology videos from YouTube	Prompting GPT-4, hand-crafted algorithms for extraction of video frames and spatial annotation	**Dataset:** Quilt-1 M^54^ **Model/Framework:** GPT-4^52^	Quilt-LLaVA^58^	**Quilt-VQA** with 985 images 1283 Q/A pairs where 940 are open-set and 343 closed-set	√
PathQABench^37^	ROI-annotated WSI and Q/A	48 H&E WSIs (25 WSIs from PathQABench-Private 23 WSIs from PathQABench-Public) + 48 close-ended Q/A 115 open-ended Q/A	PathQABench-Private private in-house cases PathQABench-Public from TCGA cases	Expert-pathologists curated and annotated	**–**	PathChat^37^	(Subsets) **PathQABench-Public** and **PathQABench-Private PathChatInstruct** 456,916 instruction-tuning dataset (Extension)	√
PathText^59^	WSI and question–text pairs	1041 WSI 9009 WSI-text	WSI and pathology report from TCGA-BRCA	Prompting LLMs, OCR, manual annotation, classifier	**–**	MI-Gen^59^	**–**	√
Prov-Path^38^	WSI and reports pairs	17,383 WSI-reports pairs	Proprietary dataset from Providence Health System (PHS)	K-means to generate four representative reports which are used to prompt GPT 3.5 to clean rest of the reports	**Model/Framework:** GPT-3.5	Prov-GigaPath^38^	**–**	√
PathCap^40^	Image-caption pairs	207 K	PubMed, internal pathology guidelines books, annotation by expert cytologists	Parsing from PubMed, image processing with YOLOv7, ConvNeXt, PLIP, caption refinement, and text processing with ChatGPT	**Dataset:** PubMed **Model/Framework:** YOLOv7,^60^ ConvNeXt^61^ ChatGPT,^62^ PLIP^42^	PathAsst^40^	**PathInstruct** 180 K pathology multi-modal instruction-following samples (Extension)	√
PathMMU^63^	Image and Q/A pairs	24,067 pathology images 33,428 Q/A	PubMed, pathology atlas, educational videos, pathologist-shared images with explanations on Twitter	Prompting GPT-4, heuristics, annotation by seven pathologists	**Dataset:** Quilt-1 M,^54^ OpenPath^42^ **Model/Framework:** GPT-4,^52^ YOLOv6^64^	**–**	PubMed, SocialPath EduContent, Atlas, PathCLS (Subsets)	√
Dataset from CONCH^41^	Image–caption pairs	1,786,362 image-caption pairs	PubMed, publicly available research articles, internal data from Mass General Brigham institution	Hand-crafted workflow with YOLOv5, CLIP, BioGPT	**Model/Framework:** YOLOv5,^65^ CLIP BioGPT^66^	CONCH^41^	**PMC-Path** (data from PubMed) **EDU** (data extracted from educational notes)	√
QUILT^54^	Image-text pairs	437,878 images aligned with 802,144 text pairs	1087 h of 4504 narrative educational histopathology videos from YouTube	Prompting LLMs, hand-crafted algorithms, human knowledge databases (UMLS), automatic speech recognition	**Dataset:** OpenPath,^42^ PubMed, LAION-5B^67^ (For Quit-1 M) **Model/Framework:** Whisper,^68^ GPT-3.5, inaSpeechSegmenter,^69^ langdetect^70^	Quilt-Net^54^	**Quilt-lM** with 1 million image-text pair with additional data from LAION, Twitter, and PubMed (Extension)	√
OpenPath^42^	Image–text pairs	208,414 image–text pairs	116,504 image–text pairs from Twitter posts, 59,869 image–text pairs from replies, 32,041 image–text pairs from LAION-5B	Pathology image classifier to exclude non-pathology images, CLIP image embeddings with cosine similarity to create PathLAION, other hand-crafted heuristics	**Dataset:** LAION-5B^67^ **Model/Framework:** CLIP,^45^ langdetect^70^	PLIP^42^	**PathLAION** 32,041 pathology images from the LAION-5B dataset (Subset)	√
Dataset from Mi-Zero^71^	Image–caption pairs	33,480 image–caption pairs	Publicly available educational resources combined with ARCH	Hand-crafted algorithms to filter out non-pathology images	**Dataset:** ARCH^72^	MI-Zero^71^	**–**	√
ARCH^72^	Image–caption pairs	11,816 image–caption pairs	PubMed and pathology textbooks	Hand-crafted algorithms with tools like PubMed Parser, Pdffigures 2.0	**Model/Framework:** PubMed Parser^73^ Pdffigures 2.0^74^	**–**	**–**	√
PathVQA^75^	Image and Q/A pairs	4998 images 32,799 Q/A pairs	Pathology textbooks, Pathology Education Informational Resource (PEIR) digital library	Hand-crafted algorithms with tools like PyPDF2, PDFMiner, Stanford CoreNLP toolkit	**Model/Framework:** PyPDF2, PDFMiner Stanford CoreNLP toolkit^76^	**–**	16,465 open-ended Q/A subset, 16,334 close-ended Q/A subset	√
4. Annotated timeline of the surveyed articles (Fig. 3), which provides a clear idea of the evolution of the FMs and VLMs in CPath. In addition, a project page is available (https://cpath-fms-vlms.github.io/survey/) with additional information about the surveyed articles.

**Fig. 3:**
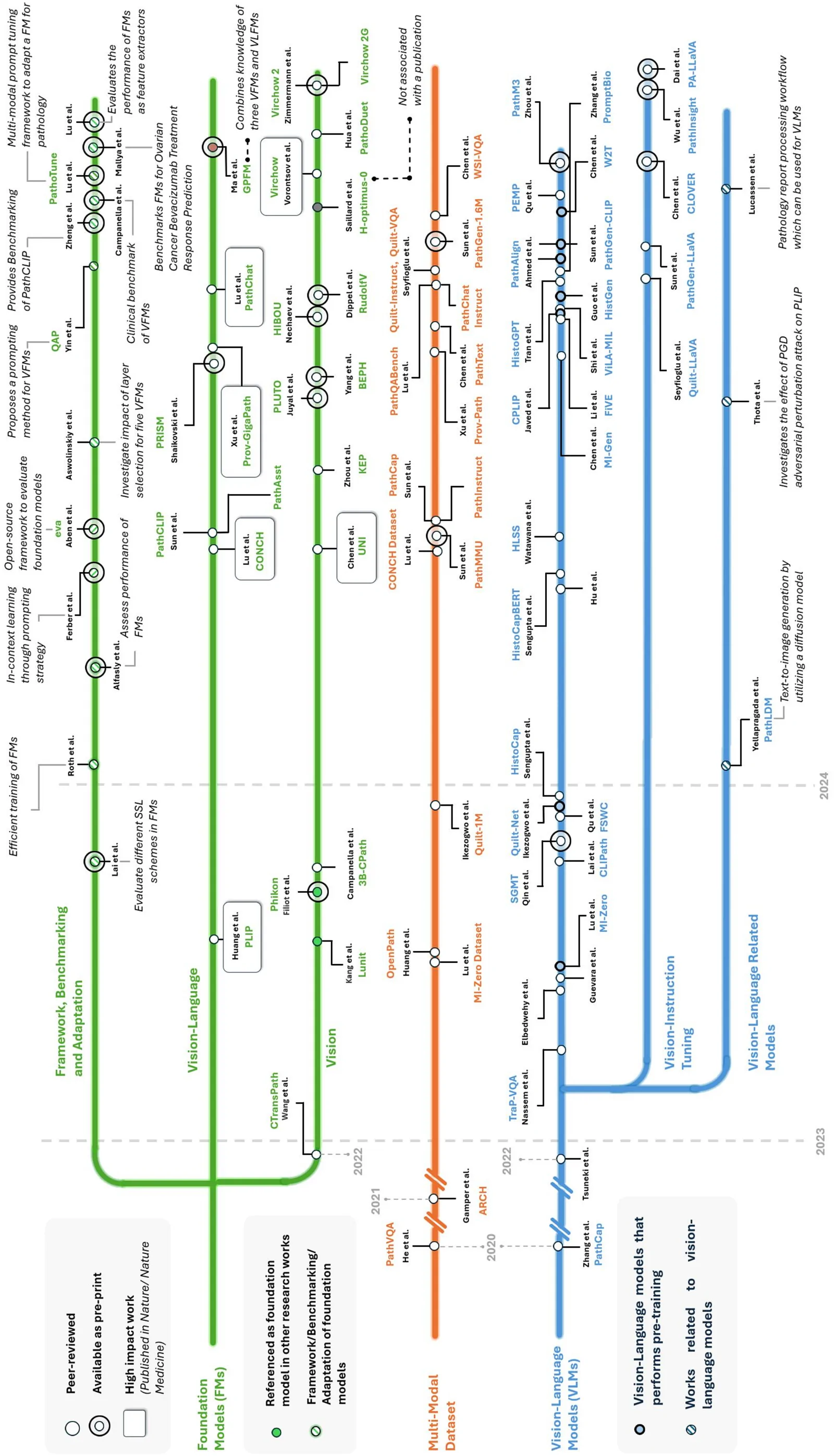
Visualization of the timeline of recently published work in CPath utilizing FMs and VLMs as well as multi-modal datasets. To maintain transparency, we clearly annotate research articles that have been peer-reviewed and articles that are available as pre-prints. Furthermore, high-impact pioneering research works published in prominent journals are highlighted. For pre-prints, if there are multiple versions, the latest version and the corresponding date are used.

The rest of the article is organized as follows: In Section 2, existing multi-modal datasets are listed along with details about their source, pre-processing techniques, etc. Section 3 outlines existing FMs in CPath and descriptions for the vision and vision-language pre-training schemes for those FMs are provided. In Section 4, an extensive list of VLMs in CPath is presented along with details of their architectures, utilized datasets, and contributions. Finally, Section 5 concludes the article.

## Multi-modal datasets in pathology

In this section, a comprehensive summary of the existing multi-modal datasets available in CPath is provided. The modalities taken into account are vision and language. All key information regarding each dataset is summarized in Table 2.

To have a comprehensive understanding of the existing multi-modal datasets for pathology, three different components need to be considered as shown in Fig. 4.

**Fig. 4:**
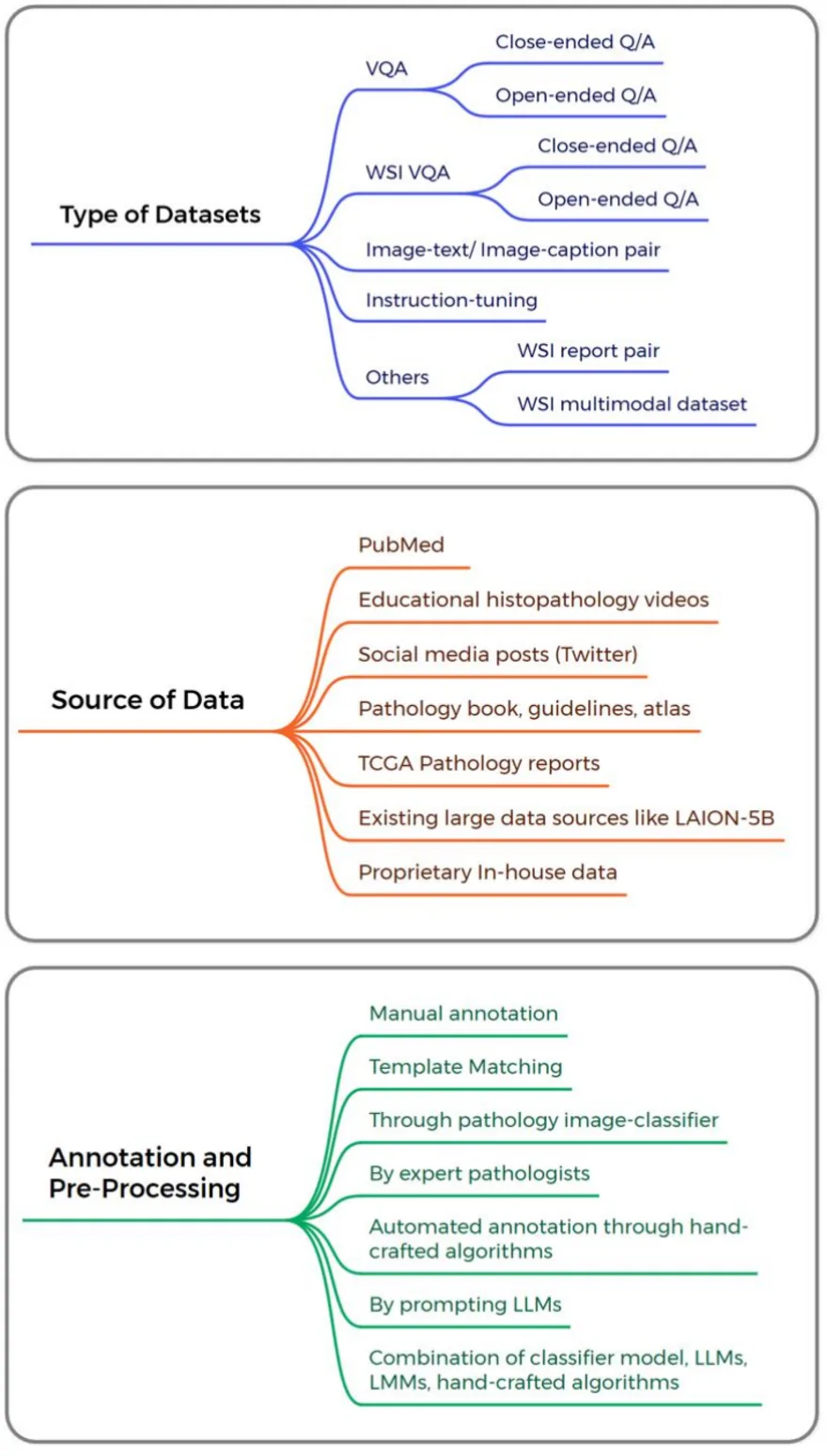
Different components of multi-modal datasets in computational pathology: Type of datasets, sources of data, annotation, and pre-processing.

### Type of datasets

The first component is the type of data, which can be broadly classified into five categories. Prov-Path^38^ fall into the last category, which is dissimilar to the rest of the datasets. The dataset for Prov-Path^38^ utilizes WSI and the corresponding reports along with histopathology findings, cancer staging, genomics mutation profiles, etc. collected by Providence Health System (PHS).

The rest of the datasets can be sectioned into the remaining four categories. The image–caption/image–text pair category (dataset from CONCH,^41^ PathGen-1.5M,^53^ Quilt-1M,^54^ PathCap,^40^ OpenPath,^42^ dataset from MI-Zero,^71^ and ARCH^72^) involves a low- or medium-quality image and an associated piece of text for that image. This text can be a short caption with a description of the image or a more elaborate description. ARCH^72^ is the earliest dataset in this category that utilized PubMed and pathology textbooks to extract the texts. PathGen-1.5M^53^ is the latest dataset in this category, but unlike other datasets in this category, the images are patches extracted from WSIs.

This is in contrast to other datasets in this category like OpenPath,^42^ which is constructed with Twitter posts and replies and Quilt-1M,^54^ which is constructed with frames extracted from educational pathology videos. However, the curation and annotation process of this category is much easier as it can be automated with hand-crafted algorithms and heuristics. However, it comes with an inevitable noisy data as due to the automated process a lot of artifacts can be present in the data.

The WSI VQA/text category (WSI-VQA,^51^ PathQABench,^37^ and PathText^59^) contains question and answer pairs or texts associated with WSIs. The most common data source is the cancer genome atlas (TCGA),^20^ which contains a large repository of WSI and patient report pairs. The VQA part can be of two types: close-ended question–answer pair and open-ended question–answer pair. Close-ended question–answer pairs are of a multiple-choice type or short-answer type which have pre-defined answers. On the other hand, open-ended question–answer pairs contain answers that are in natural language form. Among the datasets in this category, PathQABench^37^ is unique as it contains region of interest (ROI) annotation of WSIs performed by expert pathologists. It has two subsets as PathQABench-Public and PathQABench-Private. The former is publicly available as it was constructed with TCGA WSIs and reports, and the latter was constructed with in-house data.

The next category, which is VQA (PathMMU,^63^ Quilt-VQA,^58^ and PathVQA^75^) is similar to the previous category as it also contains close- and open-ended question–answer pairs, but the associated images are not WSIs but rather low- and medium-quality images. Among these datasets, PathVQA^75^ is the first research to curate a pathology-specific VQA dataset. PathMMU^63^ is the latest and largest dataset in this category and it also provides explainability annotations with each answer. Another category, which is the instruction-tuning dataset (Quilt-Instruct,^58^ PathInstruct,^53^ PathChatInstruct,^37^ and extension of PathGen-1.6M^53^) is a unique kind of dataset, as this type of dataset is used to provide conversational ability to an existing multi-modal model. The common workflow is that the instruction-tuning dataset is applied in the last phase to fine-tune an already trained VLFM or VLM. All of these datasets were created following the strategy mentioned in LLaVA^55^ or LLaVA-1.5.^77^ More details about the instruction-tuning phase are provided in sections 3.3 and 4.3.

A comparison of dataset sizes is shown in Fig. 5. Among the datasets in VQA, the largest dataset is PathMMU,^63^ for WSI-VQA and WSI-text, the largest one is PathText,^59^ for the instruction-tuning dataset it is PathChatInstruct,^37^ and finally for the image–caption pair, the largest one is the dataset from CONCH.^41^

**Fig. 5:**
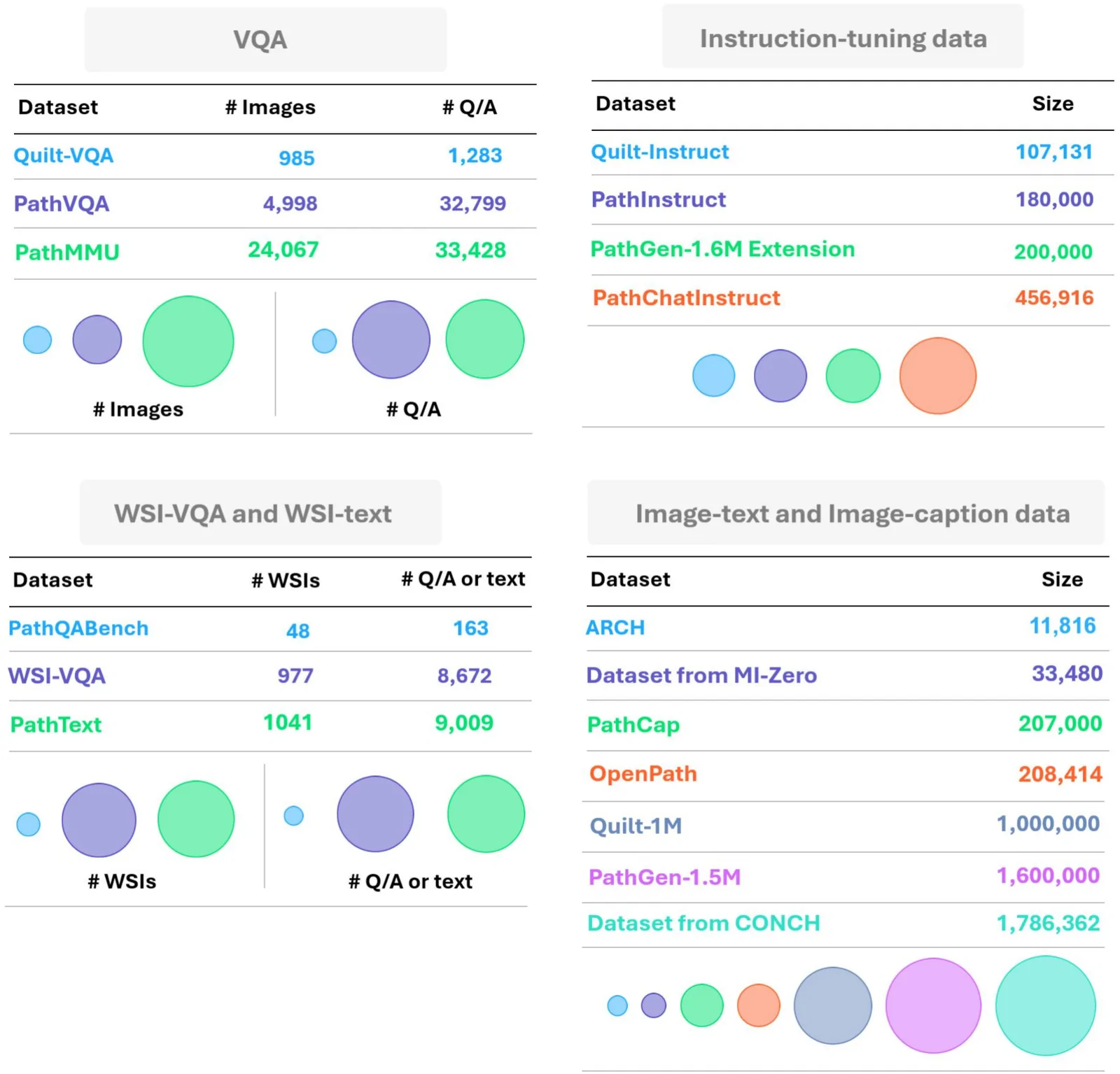
Comparison between the size of different multi-modal datasets. The size of the bubbles indicates the size of the data set (For visual clarity, the scale used for bubble size is the same within a specific group, but differs between groups).

### Source of data

The second component to consider is the source of data which largely dictates the third component, the annotation, and pre-processing. PubMed is a common data source containing pathology images and captions/text. However, the quality of the data is not as high as that of the TCGA repository that contains WSIs and corresponding pathological reports. Other high-quality data sources that contain WSIs and pathology reports are in-house proprietary datasets (PathQABench-Private, Prov-Path). A unique data source, OpenPath,^42^ contains pathology images and texts from Twitter posts and replies associated with a large pathology community.

This dataset was supplemented by pathology-specific data from the large-scale artificial intelligence open network (LAION) data repository.^67^ Pathology textbooks and Atlas are also large knowledge sources that can be used to extract image caption/text pairs. In a couple of recent research,^54^, ^58^ educational histopathology videos on YouTube are being used as the source of pathology image and text pair. However, curation of this kind of dataset requires a series of hand-crafted algorithms and many external tools. Based on the above discussion, it is apparent that there is a trade-off between the quality and volume of the data.

Another key point is that most of these datasets contain other datasets as one of the subsets. An example of that is Quilt-1M,^54^ which contains OpenPath,^42^ PubMed, and LAION^67^ as subsets in addition to the proposed Quilt dataset. A visualization of Quilt-1M^54^ and its subsets is provided in Fig. 6. Another such example is the PathMMU^63^ dataset (shown in Fig. 7), which contains five different subsets, PubMed, SocialPath, EduContent, Atlas, and PathCLS each containing data from different sources.

**Fig. 6:**
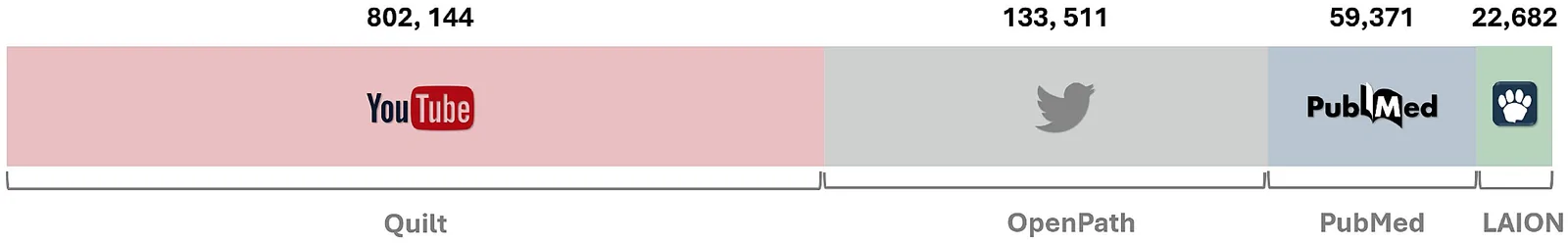
Subsets of the *Quilt-1M* dataset and the corresponding number of image–text pair for each subset.

**Fig. 7:**
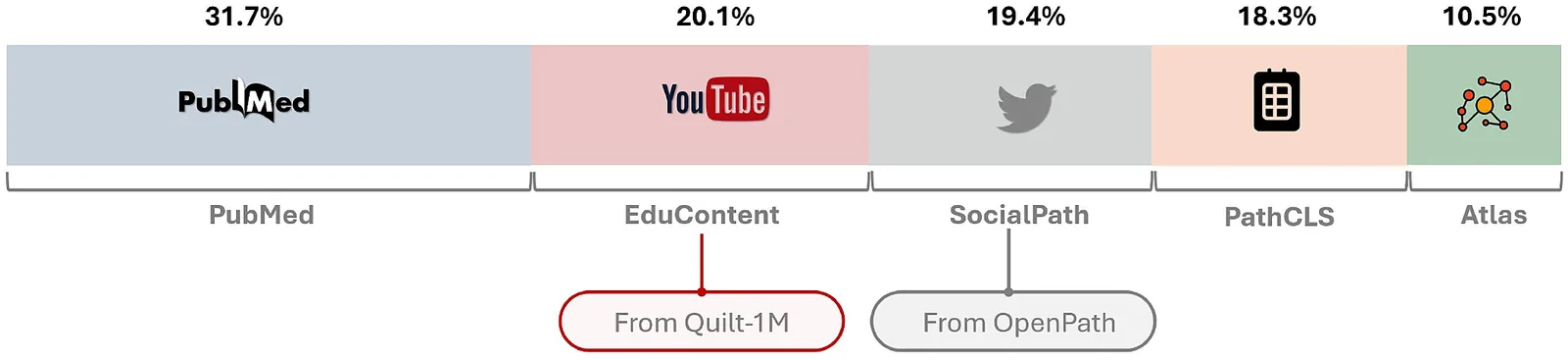
Subsets of the *PathMMU* dataset and proportion for each subset. The EduContent and SocialPath subset is sourced from Quilt-1M and OpenPath dataset.

### Annotation and pre-processing

The third component is the annotation and pre-processing steps of data curation and generation. Among the surveyed articles, all employ a series of steps depending on the type of dataset and the pre-processing pipeline is unique for every article. However, some specific steps are similar if the data source is the same. For example, all articles that utilize PubMed as a data source use some kind of parsing process^73^ to parse and extract figures and texts. In addition, they employ light-weight classifiers and object detection architectures (YOLOv5,^65^ YOLOv6,^64^ and YOLOv7^60^) to distinguish between pathology and non-pathology images, detect and separate subfigures, etc. Another common approach is to prompt LLMs to format and refine captions/text or structure extracted information according to a pre-defined template. These LLMs include generalized LLMs like GPT-4, GPT-3.5, ChatGPT, and also specialized LLMs like BioGPT.^66^ Another widely used strategy is using a trained CLIP-based model and using cosine similarity as a metric to classify pathology and non-pathology images.

Apart from the approaches mentioned above, there are a lot of other hand-crafted algorithms, heuristics, and tools, which are summarized in Table 2.

## Foundation model

In this section, an overview of existing FMs in CPath is provided. First, the characteristics of FMs (Section 3.1) are provided to remove any ambiguity for the later sections. Next, pre-training workflow and typical pre-training schemes (Section 3.2) are mentioned along with the instruction-tuning phase (Section 3.3), which is becoming a widely adopted technique to provide FMs with conversational ability. Next, the downstream tasks and the associated downstream datasets (Section 3.4) for the FMs are outlined.

### Characteristics of FMs

A model can be classified as FM if it holds the following characteristics:

1. The first characteristic that is common to all FMs is the SSPT. The data used in the pre-training phase do not have any explicit label or annotation.
2. The training goal of FMs is not to solve any specific task but rather to learn a general and rich representation space. For VFMs, it is a vision representation space and for VLFMs, it is a vision-language representation space. The training of FMs is termed as “pre-training,” as in later stages, further training is required to optimize for a specific task.
3. In CPath, FMs are trained using large and diverse datasets that encompass tissue samples from different organs and anatomic sites. In addition, some research^26^, ^29^ put effort into capturing diversity in terms of scanner types, magnification levels, stain types, preservation methods, etc. The idea is to capture the representation of a wide range of tissue and disease types. In Fig. 8, a high-level visualization is provided highlighting different aspects of FMs.

**Fig. 8:**
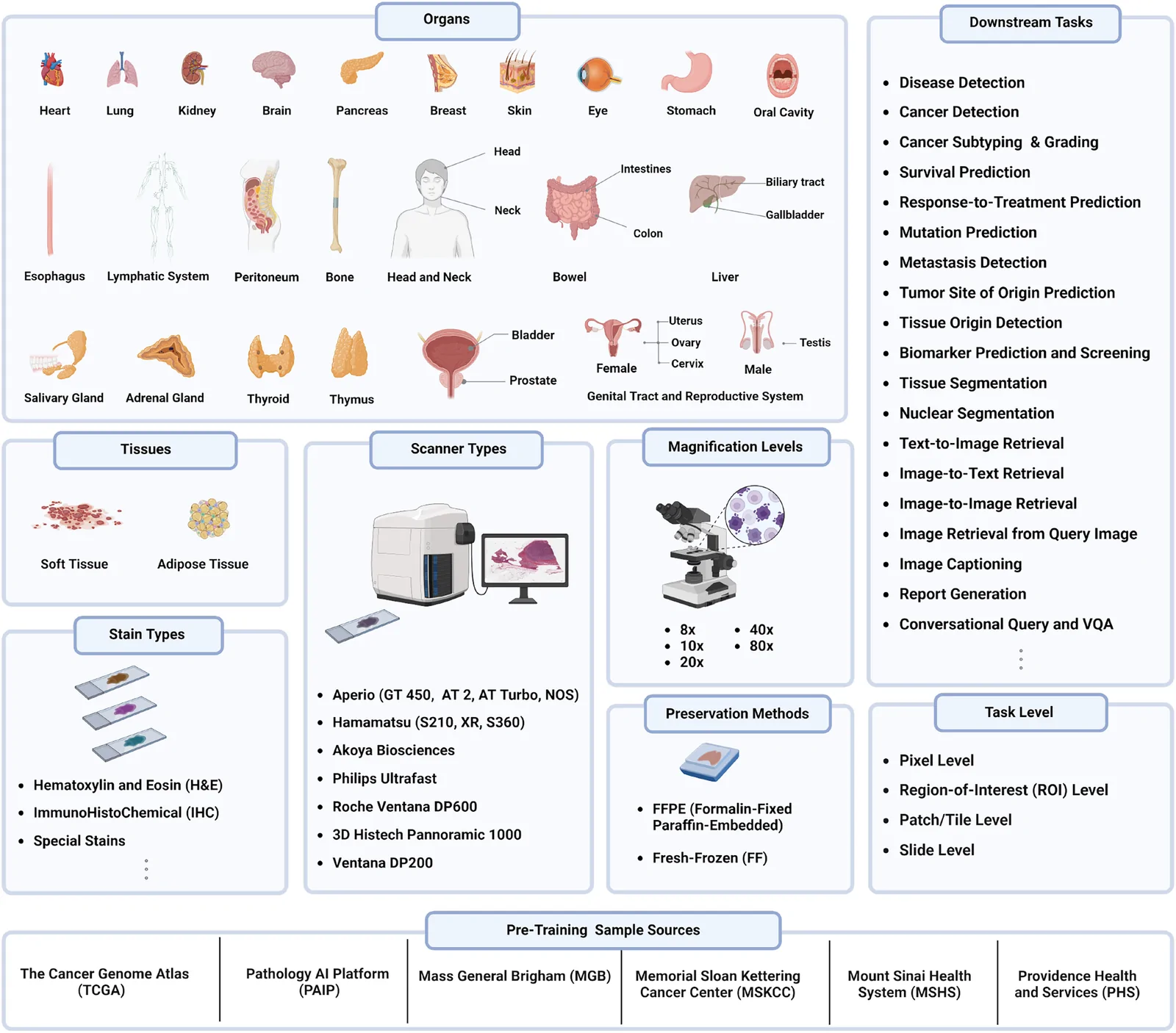
A high-level visualization of different factors of variability in terms of organs, stain types, scanner types, magnification levels, preservation methods, downstream tasks, task levels, pre-training tissue sample sources, etc. in foundation models. The research that puts the most emphasis on ensuring variability are RudolfV^26^ and PLUTO.^29^ Among the tissue sample sources for pre-training, TCGA and PAIP are publicly accessible and the rest are proprietary.
4. Another characteristic is the size of models that typically have parameters on the scale of millions. A huge amount of computing resources is put into training these models involving multiple GPUs.

As shown in Fig. 3, the FMs are sectioned into three separate categories. One category encompasses VFMs^22^, ^23^, ^24^, ^25^, ^26^, ^27^, ^28^, ^29^, ^30^, ^31^, ^32^, ^33^, ^34^, ^35^ in CPath, the second category encompasses VLFMs,^36^, ^37^, ^38^, ^39^, ^40^, ^41^, ^42^ and the last category utilizes these FMs by providing a benchmark, framework, or adapting existing FMs.^78^, ^79^, ^80^, ^81^, ^82^, ^83^, ^84^, ^85^, ^86^, ^87^, ^88^, ^89^

### Pre-training workflow and strategies

The typical pre-training workflow of FMs is shown in Fig. 9, which provides a high-level visualization of different phases in the workflow.

**Fig. 9:**
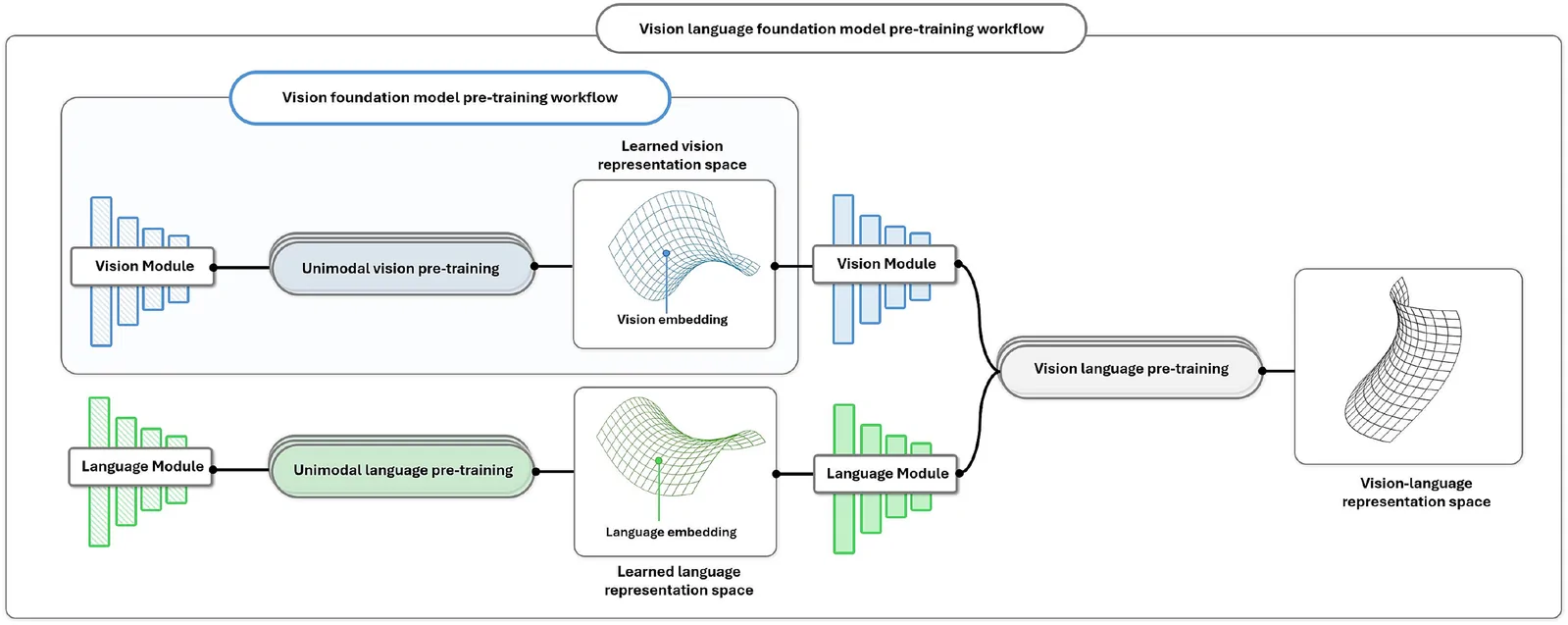
Typical pre-training workflow in FMs. In general, it starts with a vision module and a language module with randomly initialized weights or pre-set weights. VFMs only go through unimodal vision pre-training to learn a vision representation space. On the other hand, VLFMs can optionally go through unimodal pre-training for their vision and language modules.

In this single diagram, both pre-training strategy for VFM and VLFM is shown. For VFMs, a vision module with some initial weight is leveraged in pre-training. The term “vision module” is used to generally represent a wide range of vision architectures and also modified versions of these architectures with additional layers. The most common architecture is variants of vision image transformers (ViTs)^90^, ^91^, ^92^ which includes ViT—S, ViT—B, ViT-L, ViT-G, and ViT—H. These variants mainly differ in their size. ViT-S (Small) is the most lightweight version with fewer layers and parameters, making it faster and less demanding to train. ViT-B (Base) is the standard version that offers a good balance between performance and computational cost. ViT-L (Large) is a bigger model with more layers and parameters, allowing it to learn more detailed representations. ViT-H (Huge) and ViT-G (Giant) are the largest versions, offering the highest capacity but requiring significantly more memory and computing power. In general, the choice of variant depends on the size of the pre-training dataset and the available computational resources.

Though there are specialized architectures proposed in BEPH^28^ and Prov-GigaPath^38^ which uses BEiTv2^93^ and GigaPath^38^ architecture, respectively. Note that, some models initialize the architectures with ImageNet^94^ weights to get an initial vision representation space which can be transformed through pre-training. In Table 3, a summary of pre-training strategies in different phases is outlined.

**Table 3:** Different pre-training phases and corresponding SSPT strategies.

Pre-training phase	Strategy	
Unimodal vision pre-training	Self-distillation	DINO,^95^ DINOv2^96^
	Contrastive learning	MoCov2,^97^ MoCov3^98^
	Masked image modeling (MIM)	MAE,^99^ iBOT (MIM+Self distillation)^100^
Unimodal language pre-training	No specific strategy	
Vision-language pre-training	CLIP,^45^ CoCa^101^

**Table 4:** Summary of vision pre-training strategy and vision pre-training dataset of foundation models in pathology.

Model	Vision pre-training strategy			Vision pre-training dataset				Type of foundation model	Availability (*Linked*)
	Category	Approach	Architecture	Source	Size	Stain	Additional information		
GPFM^36^	MIM + Self-distillation+ (Expert knowledge distillation)	Custom	Custom architecture that uses other FMs including UNI, Phikon, CONCH	33 public datasets including TCGA, GTExPortal PAIP, CPTAC, etc	**Slides:** 86,104 **Tiles:** 190,000,000	**–**	47 data sources 34 major tissue types	Combines Knowledge of VFMs and VLFMs	√
Virchow 2^22^ Virchow 2G	Self-distillation	DINOv2	ViT-H ViT-G	Memorial Sloan Kettering Cancer Center (MSKCC) + Institutions worldwide	**Slides:** 3,134,922 **Tiles:** —	H&E IHC	225,401 patients 493,332 cases 871,025 specimens	Vision	√
PathoDuet^23^	Contrastive learning	MoCov3	ViT-B	TCGA	**Slides:** 11,000 **Tiles:** 13,166,437	H&E	**–**	Vision	√
Virchow^24^	Self-distillation	DINOv2	ViT-H	Memorial Sloan Kettering Cancer Center (MSKCC)	**Slides:** 1,488,550 **Tiles:** 2 billion	H&E	17 organs 119,629 patients 208,815 cases 392,268 specimens	Vision	√
RudolfV^26^	Self-distillation	DINOv2	ViT-L	108,433 slides from 15 labs across the EU and USA + 26,565 slides from TCGA	**Slides:** 133,998 **Tiles:** 1.2 billion	H&E (68%) + IHC (15%) + other (17%)	14 organs, 15 labs, 58 tissue types, 129 stains, 6 scanner types FFPE and FF tissue	Vision	**–**
Hibou^27^	Self-distillation	DINOv2	ViT-B (for Hibou-B) ViT-L (for Hibou-L)	Proprietary data	**Slides:** 936,441H&E + 202,464 non-H&E + 2676 cytology slides **Tiles:** 1.2 billion patches for Hibou-L, 512 million patches for Hibou-B	H&E + non H&E	306,400 cases	Vision	√
BEPH^28^	MIM	**–**	BEiTv2^93^ with VQ-KD autoencoder and ViT-B encoder	TCGA	**Slides:** 11,760 **Tiles:** 11,774,353	H&E	32 cancer types	Vision	√
PLUTO^29^	Self-distillation + MIM	DINOv2 variation + MAE	ViT-S variant FlexiViT-S^102^	TCGA + Proprietary data from PathAI	**Slides:** 195 million **Tiles:** 158,000	H&E + IHC + Other stains	More than 30 diseases. More than 12 scanners. More than 100 stain types. More than 4 M pathologist pixel-level annotation	Vision	**–**
UNI^31^	Self-distillation	DINOv2 (also MoCov3 for comparison)	ViT-L	Proprietary Data from Massachusetts General Hospital (MGH), Brigham and Women's Hospital (BWH), Genotype–Tissue Expression (GTEx) consortium	**Slides:** 100,426 **Tiles:** over 100 million	H&E	20 major tissue	Vision	√
3B-CPath^32^	MIM self-distillation	MAE, DINO	ViT-S, ViT-L	Mount Sinai Health System (MSHS)	**Slides:** 423,600 **Tiles:** 3.25 billion	H&E	3 anatomic site 2 institutions	Vision	√
Phikon^33^	MIM + Self-distillation	iBOT	ViT-S, ViT—B, ViT-L	TCGA (Three variants TCGA-COAD, PanCancer4M, PanCancer40M)	**Slides:** 6093 **Tiles:** 43,374,634 (For PanCancer40M)	H&E	16 cancer types, 13 anatomic sites, 5558 patients (For PanCancer40M)	Vision	√
CTransPath^35^	Contrastive learning	MoCov3 variation (SRCL: semantically relevant contrastive learning)	Swin Transformer	TCGA + PAIP	**Slides:** 32,220 **Tiles:** 15.6 million	H&E	32 cancer types 25 anatomic sites	Vision	√
Prov-GigaPath^38^	MIM and self-distillation	MAE, DINOv2	GigaPath^38^ (constructed with ViT and LongNet^103^)	Providence Health System (PHS)	**Slides:** 171,189 **Tiles:** 1.3 billion	H&E, IHC	30,000 patients, 28 cancer centers, 31 major tissue types	Vision language	√
CONCH^41^	MIM + self-distillation	iBOT	ViT-B backbone in image encoder	In-house dataset	**Slides:** 21,442 **Tiles:** 1.2 million	H&E, IHC, Masson's trichrome, Congo red	350 cancer subtypes	Vision language	√

**Table 5:** Summary of vision-language pre-training and instruction tuning phase of foundation models in pathology.

Model	Vision language modules			Vision-language pre-training phase		Instruction tuning phase	
	Vision module	Language module	Additional layer/Module	Architecture/Framework	Pre-training process	Instruction tuning	Process
PathChat^37^	UNI^31^ as vision encoder backbone	Pre-trained Llama 2^104^ LLM, which is a decoder-only transformer-based auto-regressive model	A multi-modal projector module to connect the outputs of the vision module to language module by projecting the visual tokens to the same dimension as the LLM's embedding space for text tokens	CoCa	Vision-language pre-training according to CoCa framework with CONCH dataset	√	LLaVA-1.5^77^ training approach **First phase:** Only the parameters of multi-modal projector is updated **Second phase:** Fine-tuned with instruction-following data
PRISM^39^	Virchow as tile encoder, Perceiver^105^ as slide-encoder	BioGPT first 12 layers	BioGPT last 12 layers as vision-language decoder	CoCa	Trained using contrastive loss and generative/captioning loss	✗	–
PathCLIP and PathAsst^40^	Image encoder with ViT-B	Text transformer as the text encoder with modifications mentioned in^106^	Fully connected layer after vision encoder to map the image embedding space to the corresponding language embedding	CLIP	Fine-tuning of CLIP model through contrastive learning using PathCap dataset	√	**First phase:** Detailed description-based part is used to train the fully connected layer connected to the vision encoder. **Second phase:** fine-tuned with instruction-following data via next word prediction
CONCH^41^	An image encoder with ViT-B backbone with 12 transformer layers, 12 attention heads followed by two attentional pooler modules	A GPT-style text encoder with 12 transformer layers	A GPT-style multimodal decoder with 12 transformer layers	CoCa	Visual-language pre-training with image–text contrastive loss and the captioning loss according to CoCa framework	✗	–
PLIP^42^	An image encoder with ViT-B	A text transformer as the text encoder with modifications mentioned in^106^	–	CLIP	Fine-tuning of CLIP model through contrastive learning using OpenPath dataset	✗	–

**Table 6:** Different types of downstream tasks and corresponding research works.

Downstream tasks	Performed By
Disease/Cancer/Tissue/Tumor/Molecular subtyping	23, 26, 28, 29, 30, 31, 35, 38, 39, 41, 42
Cancer detection	22, 23, 24, 28, 32, 35, 39, 42
Tumor detection	31, 40, 42
Cancer grading	31, 41
Image/Tissue/Tumor/Gland/Nuclei segmentation	26, 29, 31, 35, 41
Survival prediction	^28^
Text-to-image retrieval	30, 41, 42
Image-to-text retrieval	30, 41
Image-to-image retrieval	26, 31, 36, 42
Image captioning	^41^
Pattern/Tissue/Image classification	31, 40, 41
Biomarker prediction/Detection/Screening/Scoring	24, 26, 31, 32, 39
Metastasis detection	31, 33
Organ transplant assessment	^31^
Mutation detection/Prediction	31, 32, 33, 38
VQA	36, 37, 40
Report generation	36, 39
Survival analysis	22, 33, 36
Conversational agent	37, 40

**Table 7:** Comparison of evaluation strategies for downstream tasks in foundation models.

Criteria	Zero-shot	Few-shot	Simple-shot
No labeled data required	√	✗	✗
Unbiased representation assessment	√	✗	✗
Robust to example selection bias	√	✗	√
Reflects real-world low-resource scenarios	✗	√	√
Allows model adaptation to target task	✗	√	✗
Low variance across runs	√	✗	√
Fair cross-study comparison	√	✗	√
Resilient to domain gap	✗	√	✗
Low risk of overfitting	√	✗	√
No classifier training needed	√	✗	√
Widely explored in literature	√	√	✗
**Best choice when**	Measuring generalization of learned representations	Limited labeled data are available and task adaptation is needed	A lightweight and simple evaluation is preferred

**Table 8:** Evaluation datasets and the associated models utilizing the dataset along with the data source.

Dataset	Used by	Source
PanNuke	29, 42	√
HEST-1K	^22^	√
DLBCL-Morph	^22^	√
TissueNet	^35^	√
GlaS	^29^	√
CRAG	^35^	√
RenalCell	^30^	√
SkinCancer	^30^	√
UBC-OCEAN	^36^	√
Chaoyang	^36^	√
DigestPath	41, 42	√
WSSS4LUAD	30, 36, 40, 41, 42	√
PatchCamelyon (PCam)	22, 24, 26, 27, 36	√
MHIST	22, 24, 26, 27	√
BACH	28, 30, 31, 36	√
SICAP, SICAPv2	30, 41	√
CoNSeP	^24^	√
KIMIA Path24C	^42^	√
DHMC	31, 41	√
EBRAINS	31, 36, 41	√
AGGC	31, 41	√
PANDA	31, 36, 41	√
MSK-IMPACT	24, 39	√
SegPath	^31^	√
BRACS	31, 36	√
UniToPatho	31, 35, 36	√
HunCRC	^31^	√
BreakHis	28, 36	√
MSI-CRC & MSI-STAD	26, 27	√
TIL-DET	26, 27	√
MIDOG	22, 24, 35	√
CAMELYON16	23, 28, 31, 33, 35, 36	√
CAMELYON17-WILDS	22, 24, 29, 31, 33, 36	√
Kather colon	23, 28, 29, 30, 42	√
CRC100K	26, 27, 31, 36, 40, 41	
NCT-CRC-HE-100 K	24, 28, 30	
NCT-CRC-HE-100 K-NONORM	22, 24	
LC25000	28, 30, 40	√
LC25000Colon	40, 54	
LC25000Lung	40, 54	
TCGA Uniform	^31^	√
TCGA CRC-MSI	24, 31, 36	√
TCGA-TILs	22, 24, 31	√
TCGA	^33^	√
TCGA BRCA	27, 28, 30, 39, 41	
TCGA RCC	23, 27, 28, 30, 35, 41	
TCGA NSCLC	23, 27, 28, 29, 30, 35, 39, 41	

Mentioned data sources are shared through platforms like Zenodo, GitHub, and official challenge websites. TCGA dataset needs to be downloaded from the official portal.

**Table 9:** Summary of vision-language models in computational pathology.

Model	Architecture and utilized models/Frameworks	Dataset	Contribution	Availability
TraP-VQA^107^	• Question feature extraction with BioELMo^108^ and BiLSTM • Image feature extraction with ResNet-50 and CNN layers • Transformer encoder to fuse question and image feature, transformer decoder to upsample fused features	PathVQA	• Performs VQA • Provides interpretability in text domain with SHAP^109^ • Provides interpretability in image domain with GradCAM^110^	–
FSWC^111^	• CLIP for image and text feature extraction • GPT-4 to generate instance-level and slide-level prompt groups which introduce pathological prior knowledge into the model	• Camelyon 16 • TCGA-Lung • In-house cervical cancer dataset	• Performs few-shot weakly supervised WSI classification • Proposes a two-level (instance-level and slide-level) prompt learning MIL framework named **TOP** • Introduces a prompt guided instance pooling to generate slide-level feature	√
ViLA-MIL^112^	• GPT-3.5 as the frozen LLM which generates visual descriptive text for WSI at 5× and 10× resolution based on class level question prompt • ResNet-50 as the image encoder and corresponding CLIP transformer is used as the text encoder • Prototype-guided patch decoder which generates slide features and context-guided text decoder which generates text-features	• TIHD-RCC (in-house dataset) • TCGA-RCC • TCGA-Lung	• Performs WSI classification with a MIL framework that utilizes information from 5× and 10×. In addition, incorporates information from LLM generated visual descriptive text for both 5× and 10× scale WSIs • Introduces a novel prototype-guided patch decoder that progressively aggregates the patch features • Introduces a context-guided text decoder to refine the text prompt features by leveraging multi-granular image contexts	√
PathM3^113^	• ViT-G as the image encoder • Query-based transformer to fuse image embeddings of WSIs and corresponding captions • Frozen LLM FlanT5^114^ for caption generation	PatchGastric^115^	• Performs WSI classification and captioning through a multi-modal, multi-task, multi-instance learning framework • Leverages limited WSI captions during training • Develops multi-task joint learning inspired from^116^	–
HLSS^117^	• ResNet-50 as visual encoder with CLIP pre-training • Language encoder from CLIP • A positive pairing module (PPM) which consists of three parallel reshape layer followed by MLP • A cross-modal alignment (CAM) module which computes cosine similarity	• OpenSRH^118^ • TCGA	• Performs hierarchical (patient-slide-patch hierarchy) text-to-vision alignment for SSL • Proposes a positive pairing module (PPM) and a cross-modal alignment module (CAM)	√
FiVE^119^	• ResNet as image encoder following^120^ • Pre-trained BioClinicalBERT^121^ as text encoder and utilizing LoRA^122^ for fine-tuning • GPT-4 to generate fine-grained pathological descriptions based on raw pathology reports and expert inquiries • An instance aggregator module consisting of a self-attention module and a cross-attention module that fuses image instance embeddings and prompt embeddings to create bag-level feature	• Camelyon 16 • TCGA-Lung	• Incorporates a patch-samnpling strategy for optimize training efficiency of the model • Proposes a task-specific fine-grained semantics (TFS) module • Performs zero shot histological subtype classification, few shot classification and supervised classification with pre-training	√
CPLIP^123^	• Mi-Zero^71^ to identity-related prompts based on an image by utilizing similarity metric • GPT-3 to categorize and transform prompts into five variations • PILP to match the transformed prompts with corresponding images from OpenPath	• CRC100K • WSSS4LUAD • PanNuke • DigestPath • SICAP • Camelyon 16 • TCGA-BRCA • TCGA-RCC • TCGA-NSCLC	• Creates a pathology-specific dictionary using a range of publicly available online glossaries • Introduces a many-to-many contrastive learning instead of traditional one-to-one contrastive learning	√
HistGen^124^	• GPT-4 to clean and summarize reports curated from TCGA • A ViT-L model is pre-trained with 200 million patches by utilizing DINOv2 and used as feature extractor	• UBC-OCEAN^125^, ^126^ • TUPAC16^127^ • Camelyon 16 and 17 • TCGA-BRCA • TCGA-STAD • TCGA-KIRC • TCGA-KIRP • TCGA-LUAD • TCGA-COADREAD	• Performs WSI report generation, cancer subtyping and survival analysis • Develops a local-global hierarchical encoder module to capture features at different levels (region-to-slide) • Develops a cross-modal context-aware learning module to align and ensure interaction between different modalities	√
CLIPath^128^	• ResNet-50 as the vision encoder in CLIP • 4 fully-connected layer after the vision encoder • Transformer as the text encoder in CLIP	• PatchCamelyon (PCam) • MHIST	• Develops residual feature connection (RFC) to fine-tune CLIP with a small amount of trainable parameters and this also fuses the existing knowledge from pre-trained CLIP and the newly learned knowledge related to pathology-specific tasks	–
Ml-Zero^71^	• HistPathGPT: Unimodal pre-training of GPT- style transformer (same architecture as GPT 2- medium^106^) which is used as text encoder. • Additionally, BioClinicalBERT^129^ and PubMed-BERT^130^ are considered as text encoders. • Existing encoders like CTransPath^35^ (which is based on Swin Transformer^131^) or ViT-S (ImageNet initialization or pre-trained with MoCov3)	• 3 WSI datasets from Brigham and Women's Hospital (in-house dataset) named Independent BRCA, Independent NSCLC, Independent RCC • TCGA-BRCA • TCGA-NSCLC • TCGA-RCC	• Performs cancer subtyping with a VLM • Develops a custom pathology domain- specific pre-trained text encoder called HistPathGPT • Introduces 33,480 image-caption pair dataset	√
HistoGPT^132^	• The vision module is based on CTransPath • BioGPT as the language module • Image features sampled (with Perceiver Resampler^105^) from the vision modules are integrated into the language module via interleaved gated crossattention (XATTN) blocks^133^	• In-house dataset with 2 cohorts. Munich cohort with l6,000 histology samples and Münster cohort with 1300 histology samples with all samples stained with H&E	• Performs histopathology report generation that provides a description of WSIs with high fidelity • Provides interpretability map which shows which word in the generated report corresponds to which region in a WSI	√
PathAlign^134^	• PathSSL patch encoder which is based on ViT-S architecture and pre-trained following the approach in^89^ with masked siamese networks^135^ SSL scheme • Q-Former from BLIP-2^136^ as WSI-encoder • Among two variants PathAlign-R and PathAlign-G, PathAlign-G uses PaLM-2 S^137^ as the frozen LLM	• In-house de-identified dataset (DS1) of WSIs paired with reports collected from a teaching hospital. The stain type for the WSIs are H&E and IHC • TCGA	• Performs WSI classification, image-to-text retrieval and text generation	–
MI-Gen^59^	• ResNet and ViT (ImageNet initialization or hierarchical SSPTwith HIPT^138^) as visual encoder • CNN layer in the hierarchical position-aware module (PAM)	• PathText dataset which was created using and WSI and pathology report from TCGA-BRCA	• Performs pathology report generation from WSI • Additionally performs cancer subtyping and biomarker prediction	√
	• Transformer as encoder-decoder (both vanilla transformer and Mem-Transformer^139^). Additionally, CNN-RNN^140^ and att-LSTM^141^ is used for comparison		• Develops a hierarchical position-aware module (PAM) • Introduces PathText dataset which contains 9009 WSI-text pairs	–
W2T^51^	• ResNet-50, ViT-S pre-trained with DINO or scheme followed in HIPT^138^ as visual encoders to extract patch features • Text Encoders: PubMedBERT^130^ and BioClinical-BERT^129^ • Co-attention mapping in the decoder to align visual and text features	• WSI-VQA	• Curates a WSI VQA dataset called WSI-VQA with 977 WSIs and 8672 Q/A • Performs VQA with the proposed WSI-VQA dataset • Co-attention mapping between word embeddings and WSIs are used for interpretability	√
PathGen-CLIP^53^	• OpenCLIP framework is utilized to train a pathology- specific CLIP model • The vision encoder of LLaVA v1.5 is replaced with the trained pathology-specific CLIP model • PathGen-CLIP is combined with Vicuna^56^ LLM	• PathGen-1.6 M (for pre-training) • PatchCamelyon (*Pcam*) • CRC-100 K • SICAPv2 • BACH • Osteo • SkinCancer • LC25000 • Camelyon 16 and 17	• Curates a large-scale dataset PathGen- 1.6 M with 1.6 million image-caption pairs • Develops a pathology-specific large multimodal modal with the capability to adapt to various downstream task	√
Quilt-Net^54^	• CLIP model (based on OpenCLIP framework) • ViT-B as the image encoder • GPT-2^106^ and PubMedBERT^130^ as text encoder	• Quilt-1 M (for pre-training) • PatchCamelyon (PCam) • NCT-CRC-HE-100K • SICAPv2 • Databiox • BACH • Osteo • Renal Cell • SkinCancer • MHIST • LC25000	• Curates a large-scale dataset Quilt-1M • Performs zero-shot, linear-probing task and also cross-modal retrieval task • Provides cross-modal attention mask with Grad-CAM	√
Guevara et al.^142^	• CLIP model • HIPT as image encoder • A transformer encoder to project encoded image embedding to match the dimension of text embedding • PubMedBERT^130^ as text encoder • For the caption model pre-trained Bio GPT 2 is used • GPT-3.5-turbo^143^ to clean and refine captions. Additionally, the captions were machine-translated from two languages to English using the approach in^144^	• Self-curated dataset of WSIs and captions. WSIs are of colon polyps and biopsies. The captions contain possible 5 diagnostic labels normal, hyperplasia, low-grade dysplasia, high-grade dysplasia, or adenocarcinoma	• Performs WSI classification and caption generation through the use of weakly supervised transformer-based models	–
Hu et al.^145^	• ResNet-50 pre-trained with BYOL^146^ SSL scheme as patch encoder • Anchor-based module as WSI encoder based on^147^ which uses kernel attention transformer • Prompt-based text encoder which utilizes self-attention structure in BERT^148^	• GastricADC • In-house gastric dataset containing 3598 WSIs and reports dataset	• Performs cross-modal retrieval tasks, which includes image-to-image retrieval, image-to-text retrieval, text-to-image retrieval and text-to-text retrieval • Proposes a histopathology language-image representation learning framework • Develops a prompt-based text representation learning scheme	√
PEMP^149^	• CLIP as the backbone of the proposed model • A self-attention layer • A attention pooling layer	• In-house datasets • TCGA-CESC	• Performs survival analysis, metastasis detection and cancer subtype classification • Proposes a mechanism to introduce vision and text prior knowledge in the designed prompts (both static and learnable) at both patch and slide levels • Develops a self-attention layer called a lightweight messenger and an attention pooling layer called summary layer	–
HistoCap^150^ and HistoCapBERT	• ResNet-18 to encode thumbnail image • LSTM decoder according to^141^ as caption generator • Pre-trained HIPT encoder for WSI encoding • Trainable attention layer after HIPT encoder • In HistocapBERT the LSTM decoder is replaced by a BioclinicalBERT^129^	• Dataset from Genotype-Tissue Expression (GTEx) portal	• Performs caption generation given a thumbnail image and a WSI image	√
PathCap^151^	• ResNet-18 to encode thumbnail image • ResNet-18 to encode WSI patches • Trainable attention layer after the ResNet-18 used to encode WSI patches • LSTM as caption generation module	• Dataset from Genotype- Tissue Expression (GTEx) portal	• Performs caption generation of histopathology images using multi-scale view (thumbnail of WSIs and WSIs)	√
Elbedwehy et al.^152^	• Vision encoders include VGG,^153^ ResNet, PVT,^154^ Swin-Large,^131^ ConvNexT-Large^61^ • Language decoders and pre-trained word embedding models include LSTM, RNN, Bi-directional RNN, BioLinkBERT-Large^155^	• PatchGastric	• Performs caption generation of WSIs	–
PromptBio^156^	• PLIP as coarse-grained pathology instance classifier which filters out patches given a patch and a prompt (keeps patches associated with cancer- associated stroma) • IBMIL^157^ to encode patches followed by a fully-connected layer • GPT-4 to generate pathology descriptions • Series of transformer and MLP layer which performs biomarker prediction	• TCGA-CRC • The Clinical Proteomic Tumor Analysis Consortium (CPTAC) CRC dataset	• Performs biomarker prediction	√
SGMT^158^	• ResNet-18 (pre-trained on ImageNet) as patch encoder • Transformer encoder to encode the patch features • Transformer decoder as caption generation module	• PatchGastric	• Performs caption generation of WSIs • Proposes a novel mechanism to use subtype prediction as a guiding mechanism for the caption generation task • Develops a random sampling and voting strategy to select patches	–
Tsuneki et al.^115^	• EfficientNetB3^159^ and DenseNet121^160^ (pre-trained on ImageNet) as image encoder • Average and global pooling layer after the image encoders • RNN decoder layer for caption generation	• PatchGastric	• Performs caption generation of WSIs • Curates a captioned dataset of 262,777 patches from 991 WSIs	√

**Table 10:** Summary of pre-training strategies in VLMs.

Strategy	Reference
Performs pre-training from scratch (vision or language module)	53, 54
Utilize domain-specific vision module (HIPT,^138^ CTransPath,^35^ etc.) or language module (PubMedBERT,^130^ BioClinicalBERT,^129^ etc.)	51, 59, 132, 142, 145, 150, 156
Initialize with out-of-domain encoders (ImageNet pre-training, pre-trained CLIP, etc.)	115, 117, 152, 158

The term “unimodal” is used to signify the pre-training phase utilizing a single modality out of vision and language. This is inherently different from vision-language pre-training strategies, which involve both vision and language modalities to learn a joint vision-language representation space. In the unimodal vision pre-training phase, there are three strategies commonly used in CPath which are self-distillation, contrastive learning, and masked image modeling (MIM) approach. Each approach has its own advantages and disadvantages.

The work done in Lunit^34^ benchmarks DINO,^95^ MoCov2,^97^ SwaV,^161^ and Barlow Twins^162^ but concludes there is no clear best SSPT scheme. However, the authors conclusively prove that SSPT always outperforms ImageNet initialization.

#### Unimodal vision pre-training

The self-distillation with no label approach uses a student–teacher network to learn a rich vision representation space as shown in Fig. 10. From a single image patch, two different views are generated by applying an augmentation sampled from a set of possible augmentations (color jittering, Gaussian blur, polarization, etc). The generated output of both the networks is utilized to compute a cross-entropy loss, which is then used to update the parameters of the student network. The parameter of the teacher network is then updated through an exponential moving average of the student network parameters. Among the surveyed articles, Virchow2,^22^ Virchow,^24^ RudolfV,^26^ Hibou,^27^ and UNI^31^ use DINOv2^96^ as the self-distillation approach. PLUTO^29^ takes a unique approach by integrating MAE and a Fourier loss term to get a custom variation of DINOv2.^96^

**Fig. 10:**
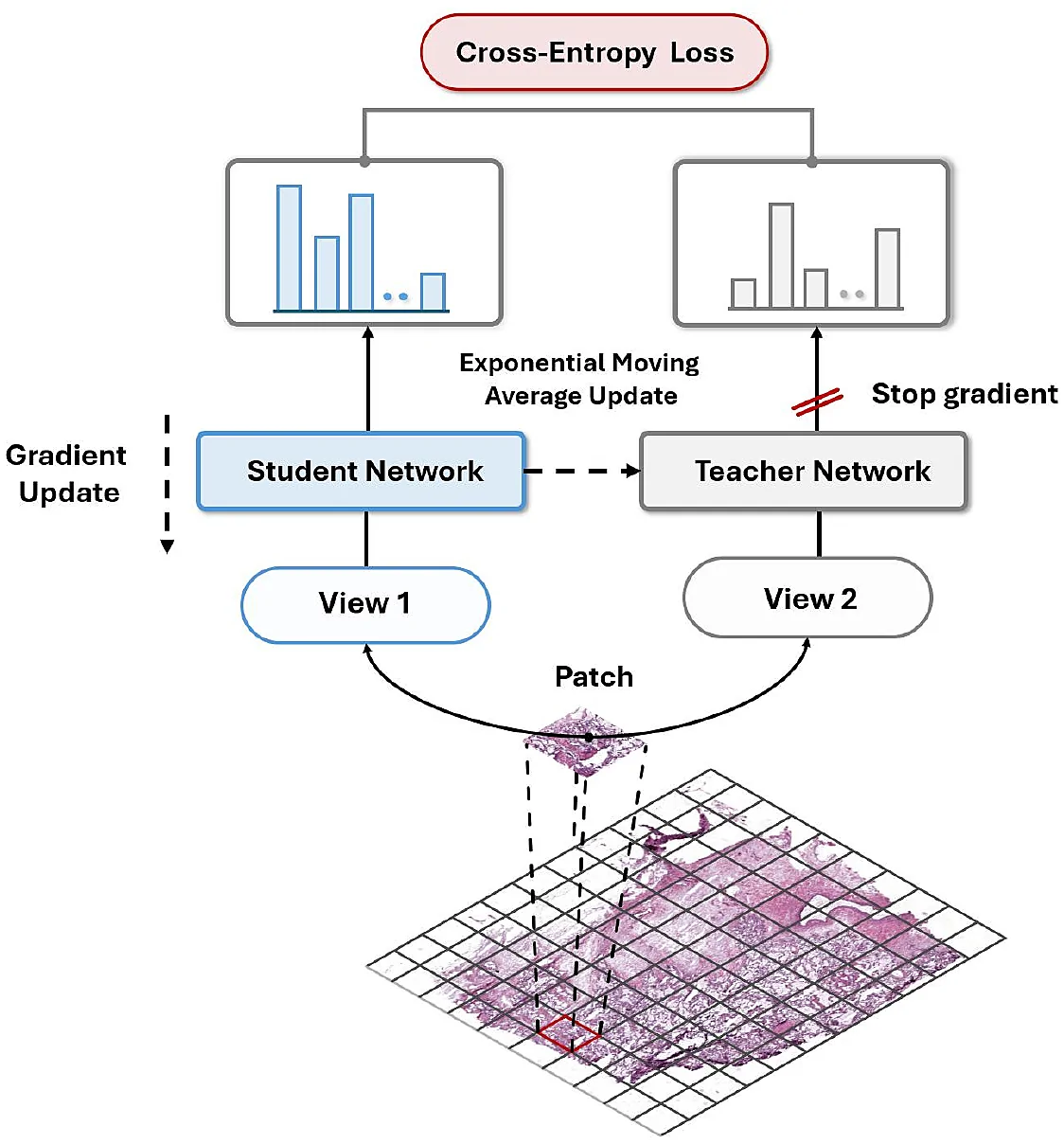
Visualization of the self-distillation approach for unimodal vision pre-training scheme.

The second popular SSPT approach is the MIM (visualized in Fig. 11) which has variants like MAE^99^ and iBOT^100^ used in FMs. In the MAE approach, randomly selected high portions of the image are masked out and the patches that are not masked out are passed through an encoder which generates latent representations of those patches.

**Fig. 11:**
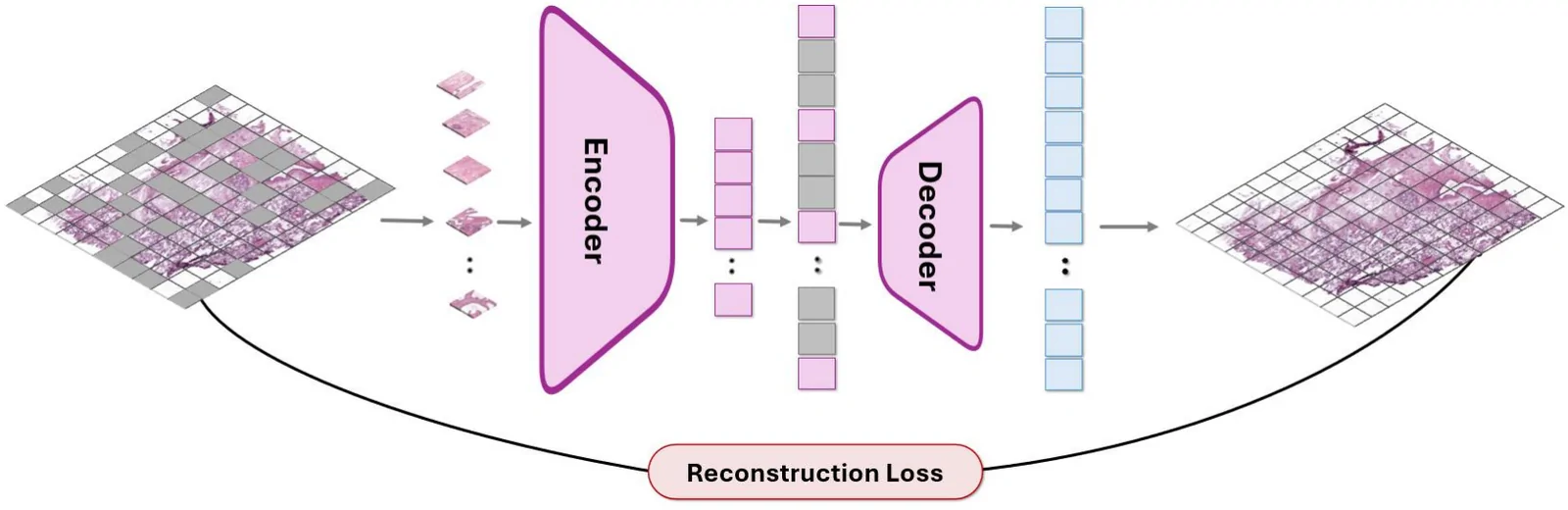
Visualization of the masked image modeling approach for unimodal vision pre-training scheme.

Then, those representations are passed through a decoder along tokens of masked out regions to reconstruct the image and reconstruction loss is used to train the model. The iBOT^100^ approach also uses MIM but adds self-distillation technique by leveraging a student–teacher network. The teacher network works as an online tokenizer and the student network learns to predict masked patches with the help of distilled knowledge of the teacher network.

Among the surveyed articles, 3B-CPath^32^ and Prov-GigaPath^38^ use MAE but in conjunction with DINO and DINOv2, respectively. Research works utilizing iBOT approach include Phikon^33^ and CONCH.^41^

Another SSPT approach that is comparatively less popular in FMs for CPath is the contrastive learning framework proposed in MoCo^163^ (visualization in Fig. 12). Over the years, variations of the proposed approach in MoCo in terms of architectural change and training blueprint have been done and MoCov2^97^ and MoCov3^98^ are the results of that.

**Fig. 12:**
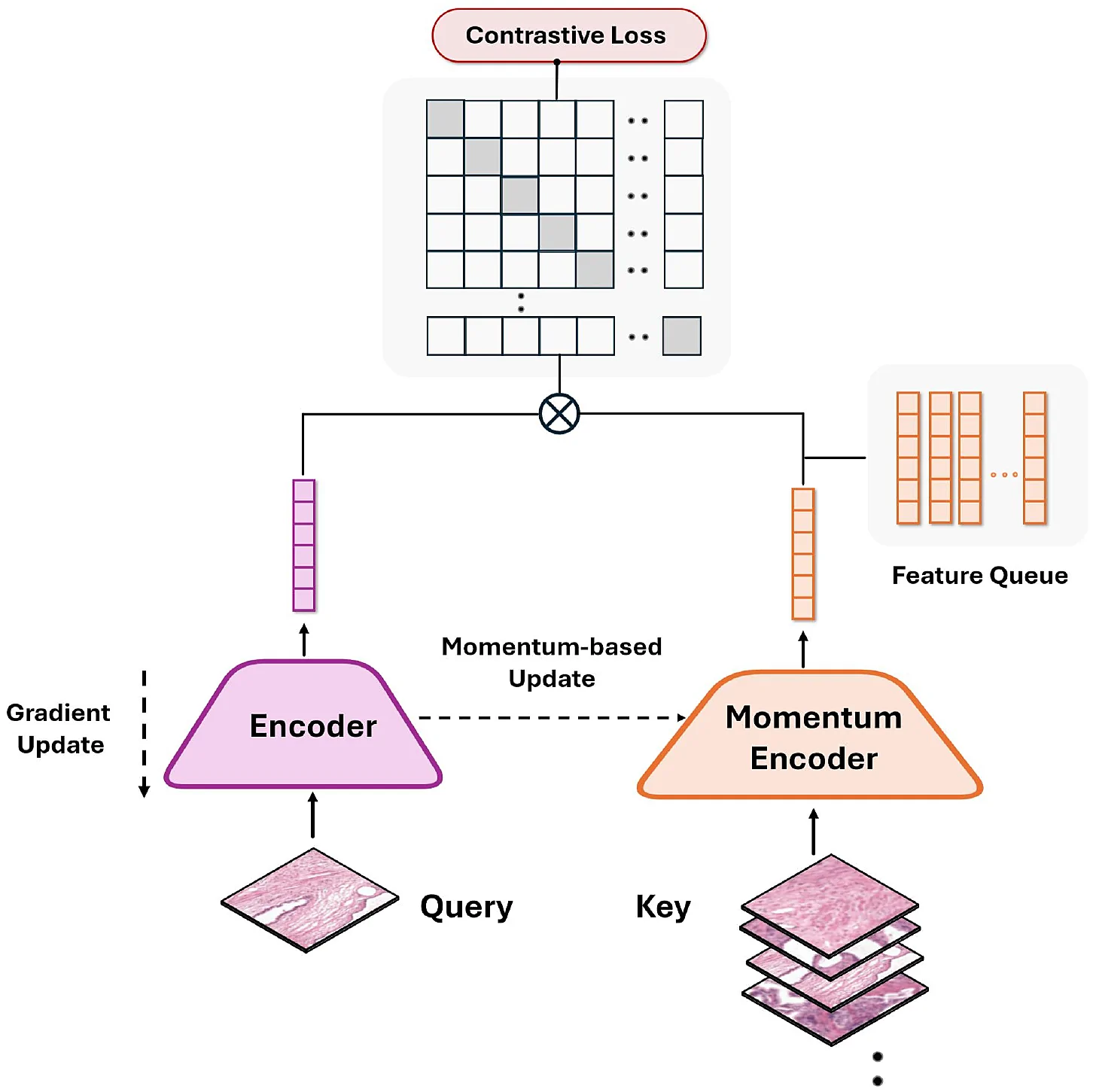
Visualization of the MoCo approach for unimodal vision pre-training scheme.

Like the self-distillation approach, MoCo also utilizes two models; one is an encoder (with query patch as input) and the second one is a special momentum encoder (with key patches as input). The embeddings generated through the encoder and the momentum encoder are used to compute a similarity score which in turn is used for contrastive loss computation. The computed loss is used in backpropagation to update the parameters of the encoder. The parameter of the momentum encoder is updated through a momentum-based update rule that utilizes the parameters of the encoder.

Another innovation of MoCo was introducing a feature queue which ensures a large set of negative samples without holding the entire dataset in memory. This also ensures the negative samples do not go stale over the training period and the model sees a diverse set of negative samples to learn a better vision representation. Among the surveyed articles, PathoDuet^23^ uses MoCov3, UNI^31^ uses MoCov3 for comparison, and CTransPath^35^ performs a unique variation of MoCov3 called semantically relevant contrastive learning. GPFM^36^ is distinct from other approaches as it includes a novel expert knowledge distillation in addition to MIM and self-distillation by utilizing existing FMs UNI,^31^ Phikon,^33^ and CONCH.^41^

#### Unimodal language pre-training

The unimodal language pre-training phase is intended to learn a rich language representation space that meaningfully captures the semantic association between words. As an example, consider two words “carcinoma” and “adenocarcinoma”, both of which refer to types of cancerous tumors arising from epithelial tissue. Adenocarcinoma is a subtype of carcinoma, specifically arising from glandular epithelium. Now, consider a third word “inflammation,” which is semantically different from the first two words. If a language module pre-trained on a large medical/pathology text corpus is used, the generated embeddings will capture this semantic information and indirectly boost the performance of the model.

Most VLFMs do not perform large-scale unimodal language pre-training. This is mainly due to the large training overhead and extensive pre-processing steps required for the text corpus. Instead, they rely on already pre-trained in-domain (trained with medical corpus) LLMs like BioGPT^66^ or other LLMs like Llama 2,^104^ GPT-variants, etc. One exception is CONCH,^41^ which pre-trains the language module with 550,000 surgical pathology reports from Massachusetts General Hospital and over 400,000 select histopathology-relevant PubMed abstracts. Using a pre-trained language module avoids the training overhead but still learning semantically meaningful embeddings.

#### Vision-language pre-training

After unimodal pre-training for the image module and text module, VLFMs might go through vision language SSPT to align or learn a joint vision-language representation space. Another interpretation is to enable the model to learn cross-modal representations that align visual features with corresponding language tokens. This phase brings together the representation learned during the unimodal pre-training steps.

The most popular vision-language SSPT approach in CPath is the CLIP approach shown in Fig. 13.

**Fig. 13:**
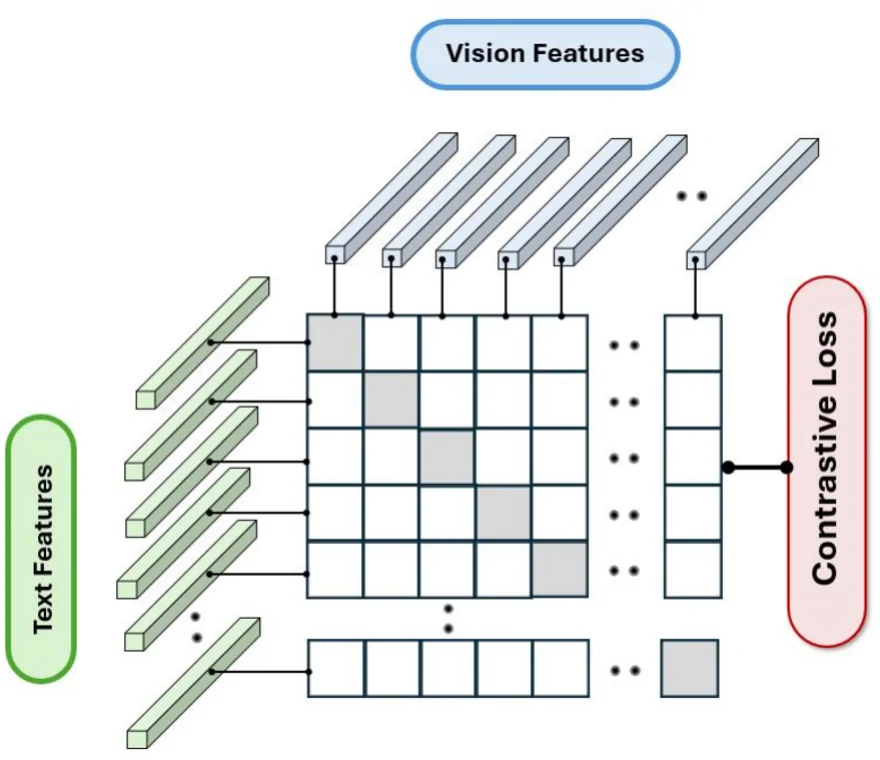
Visualization of the CLIP approach for the vision-language pre-training scheme.

The inputs to CLIP are a batch of image features encoded by an image encoder and a batch of text features encoded by a text encoder. The model is trained to pull together the corresponding image feature and text feature in the representation space and push apart the rest of the features, i.e., utilizing a contrastive objective. In other words, maximizing the similarity (specifically cosine similarity) between the corresponding image and text pair while minimizing the cosine similarity with other non-matching embeddings in the representation space.

The second widely used approach is the contrastive captioner (CoCa)^101^ framework shown in Fig. 14. It contains three different modules; an image encoder, an unimodal text encoder, and a multi-modal text decoder. It is trained by leveraging both captioning loss, which follows a generative objective and contrastive loss, which follows a contrastive objective. The input image patches are passed through an image encoder to generate image embeddings, which is used to provide cross-attention to the multi-modal text decoder. The combination of generative and contrastive objectives forces the model to learn rich vision-language representation space.

**Fig. 14:**
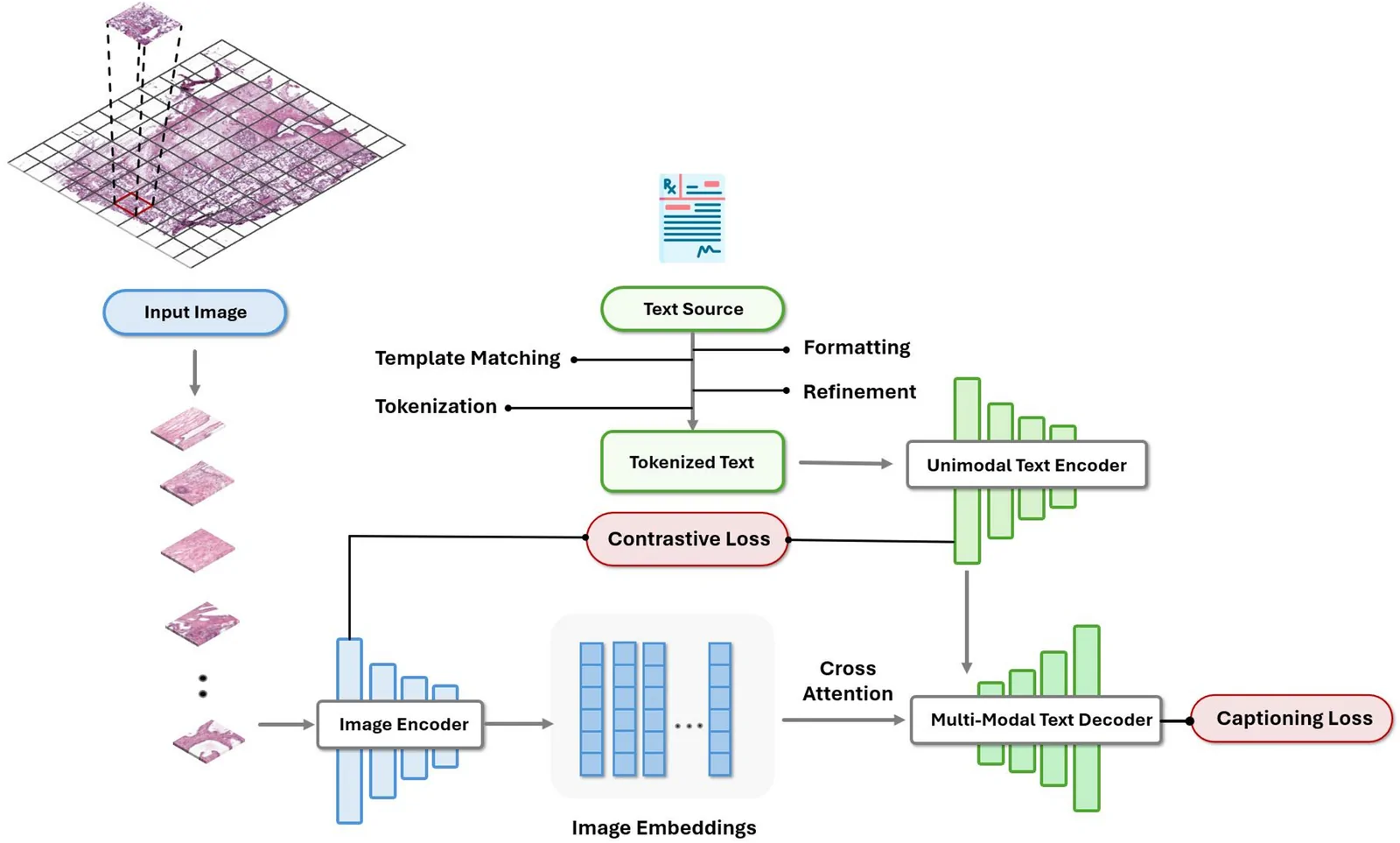
Visualization of the CoCa approach for the vision-language pre-training scheme.

Details of vision modules, language modules, additional modules, and specifics of the pre-training process of FMs are outlined in Table 5.

One recent work KEP^30^ proposes an entirely new approach in visual-language SSPT named knowledge enhanced pre-training by utilizing PathKT, a pathology knowledge tree curated by the same research. This is different from the CLIP and CoCa pre-training techniques as it uses a novel knowledge encoder and knowledge distillation technique.

### Instruction-tuning phase

For CPath, the instruction-tuning phase provides the model with conversational ability i.e. a user can prompt the model and the model will respond according to the prompt. Visual-instruction tuning adds the ability to provide an image in addition to natural language user prompts. Among the surveyed articles in FMs, PathChat^37^ and PathAsst^40^ perform instruction-tuning with self-curated datasets PathChatInstruct and PathInstruct, respectively. Both works adopt the strategy employed by LLaVA^55^ and LLaVA-1.5,^77^ which are the pioneering work in visual instruction tuning. A summary of the instruction tuning process is provided in Table 5. For both PathChat^37^ and PathAsst,^40^ the instruction tuning phase is subdivided into two phases. For both models in the first phase, the parameters of the vision encoder are frozen and the layer/module connected to the vision encoder (for PathChat,^37^ it is a multimodal projector, for PathAsst,^40^ it is fully connected layers) is trained. In the second phase, the model is fine-tuned with instruction-following data. More details about visual instruction tuning and a description of research work exclusively focusing on this aspect in CPath are provided in Section 4.3.

### Downstream tasks and datasets

In this section, several adaptation strategies (linear probing, KNN probing, and fine-tuning) and evaluation strategies (zero-shot, few-shot, and simple-shot) for FMs are explained in detail. A summary of these adaptations and evaluation strategies is summarized in Fig. 16.

FMs have the ability to adapt to a vast array of tasks by utilizing the representation space learned during SSPT. Note that, in the pre-training phase, FMs were never trained for any of these specific tasks. At the end of the pre-training of FMs, one of the following strategies is adopted to perform a specific task.

1. *Linear probing:* This is a commonly used technique where a linear classifier/regressor is trained on top of the pre-trained model. During the training of the linear layers, the parameters of the pre-trained model are kept frozen. Depending on the specifics of the tasks, the corresponding loss function and update rule are determined. This is a computationally cheap way to adapt to a downstream task as the parameters of the pre-trained model do not need to be updated.
2. *KNN probing:* This is yet another approach to adopt a FM for a specific downstream task by utilizing K-nearest neighbors algorithm. In KNN, probing a specific representation from learned representation space (through SSPT) is used to find its nearest neighbors.
3. *Fine-tuning:* This is similar to linear probing as a classifier/regressor is added on top of the pre-trained model, but the major difference is the parameter of the pre-trained models is also updated during fine-tuning. Hence, it is computationally much more costly compared to linear probing. This is sometimes also referred to as the supervised training phase. Note that, most of the time annotation is only available on slide-level. Hence, if the slide-level label is utilized it is called weakly supervised training. To attain the slide-level representation from the trained model (which is trained to generate patch-level embeddings), typically a aggregator model is used. A visualization of attaining the slide-level representation from the patch embeddings is shown in Fig. 15.

**Fig. 15:**
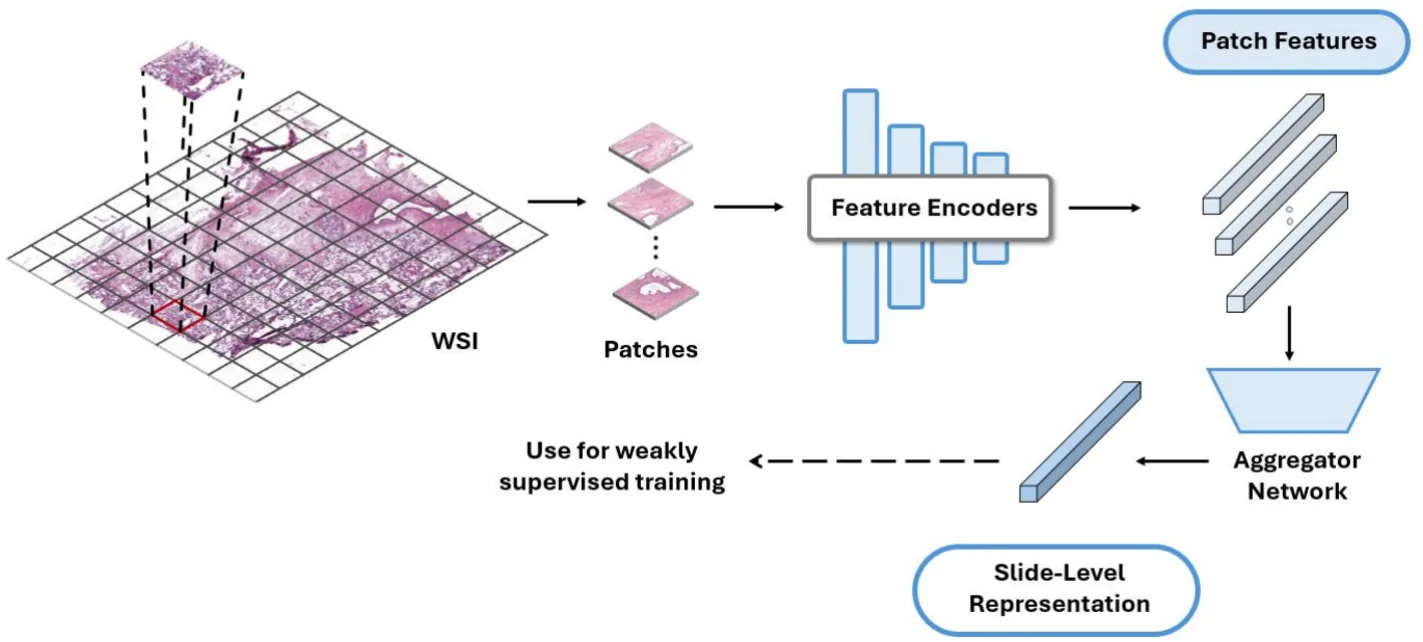
Aggregate patch-level features through an aggregator network to get a slide-level representation and use it for weakly supervised training.

**Fig. 16:**
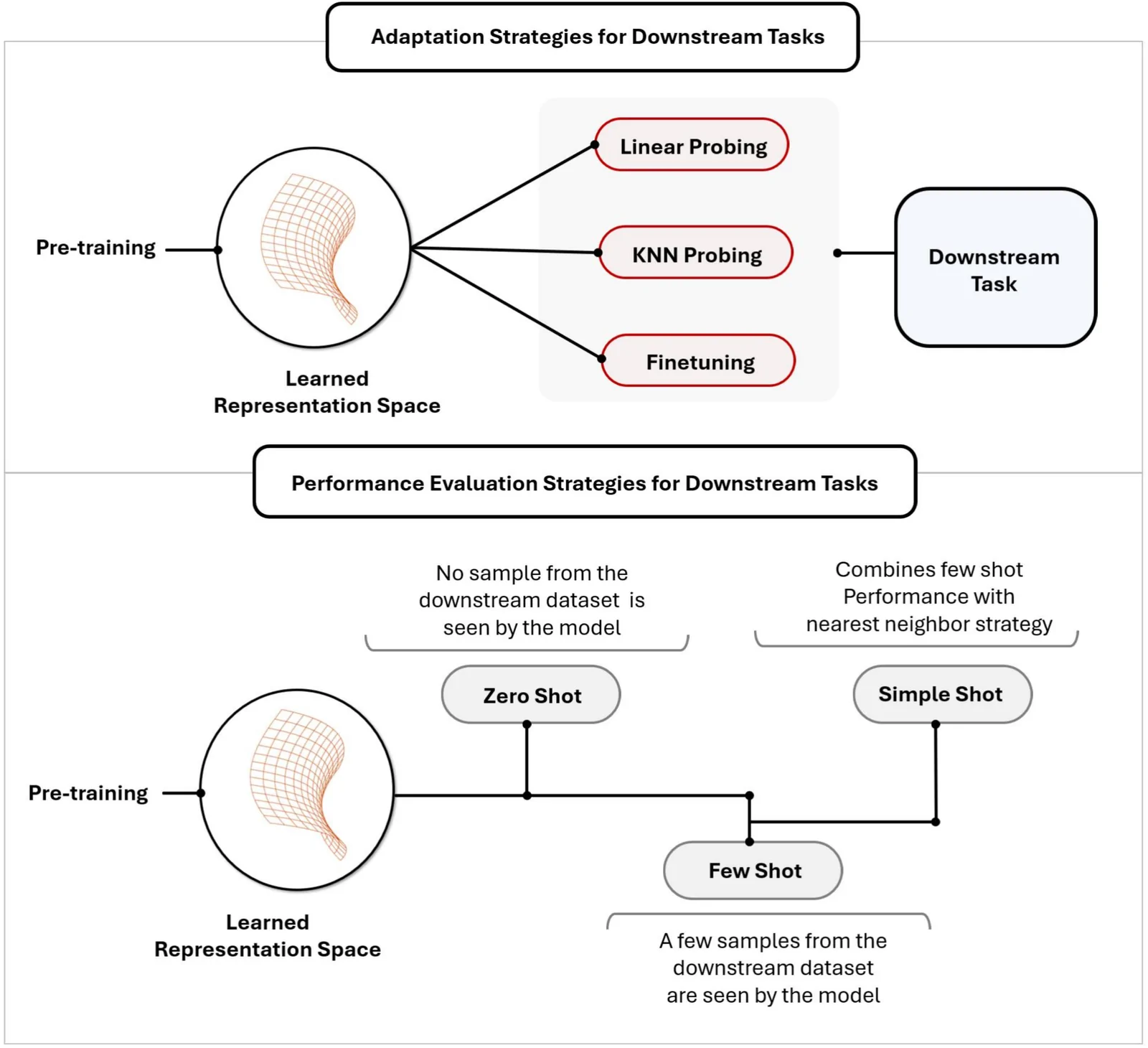
Adaptation and performance evaluation strategy for downstream tasks.

**Fig. 17:**
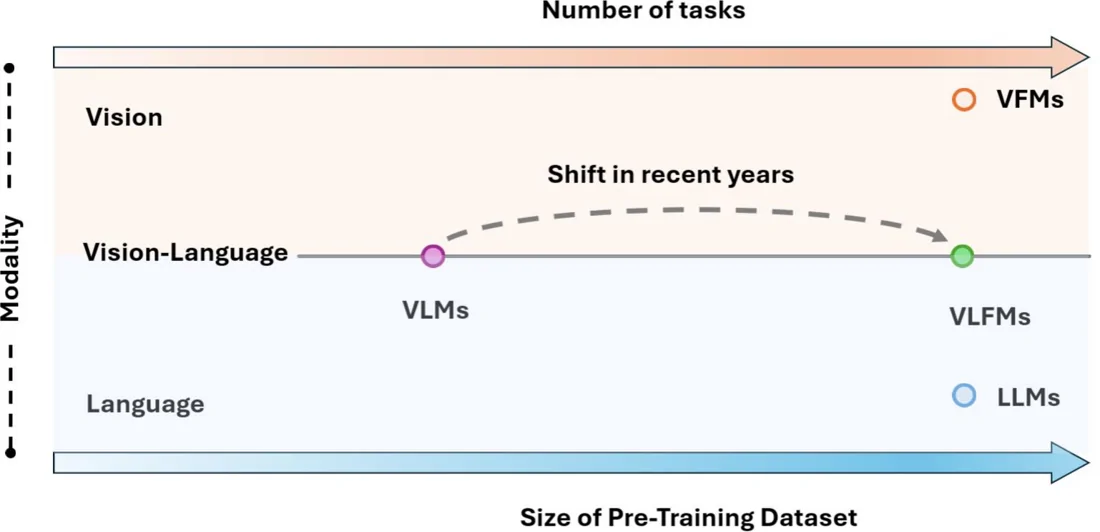
Visualization of categorization of VLMs and VLFMs. There are no clear distinctive criteria for a model to be classified as a VLFM or a VLM.

**Fig. 18:**
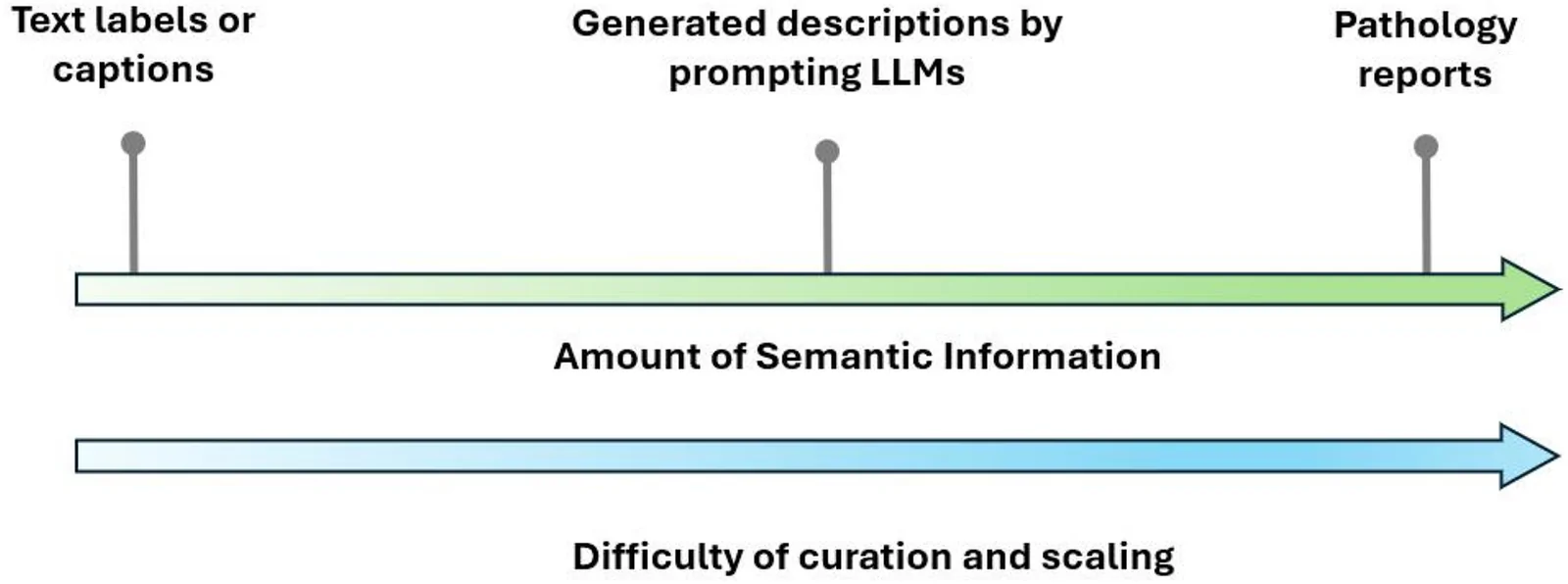
Amount of semantic information available through different text sources.

**Fig. 19:**
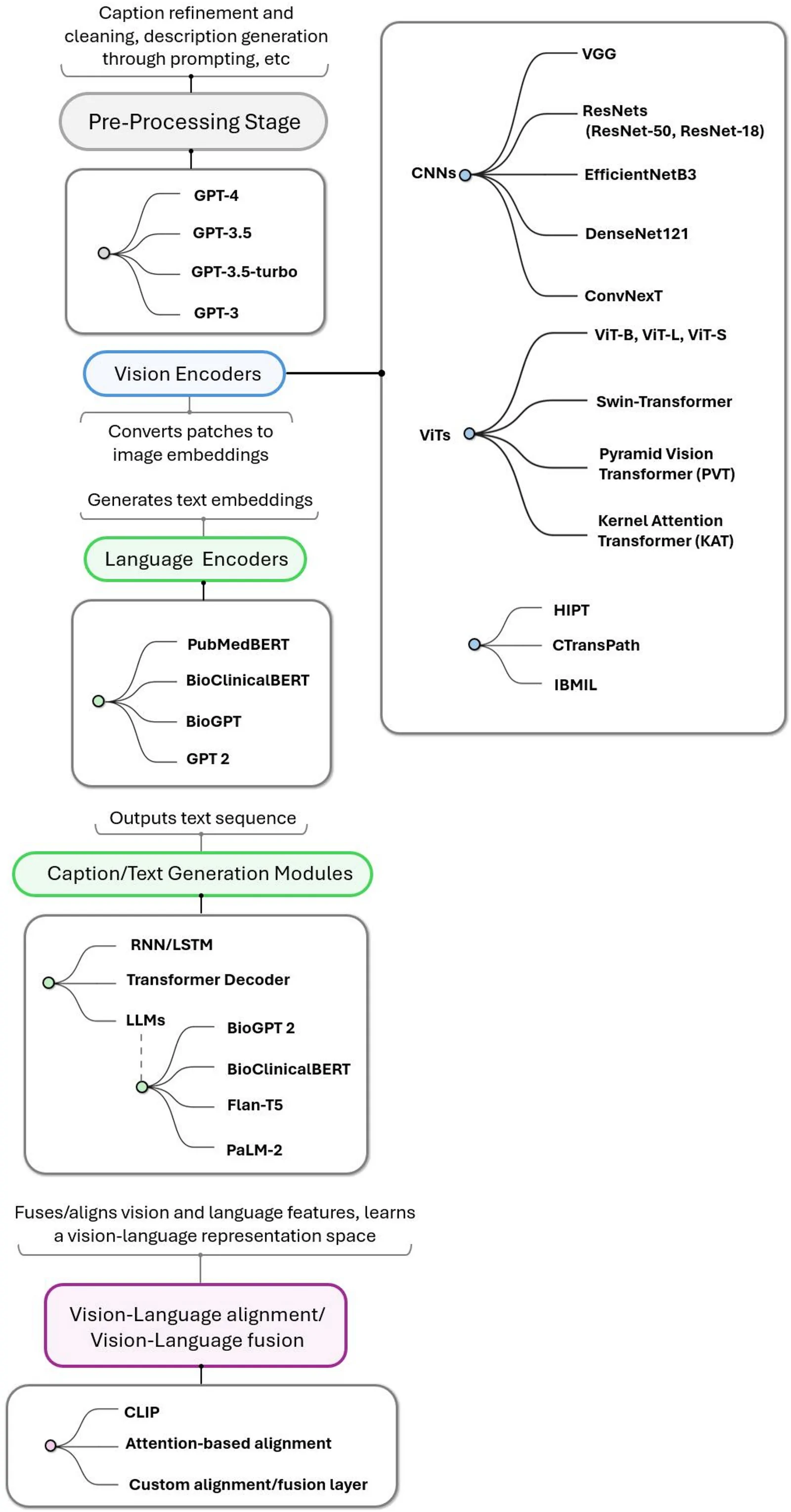
Common modules used in different stages of VLMs. The last stage is the vision-language alignment or fusion stage where the vision and language embeddings generated from the earlier stages are combined.

In Table 6, a summary of downstream tasks is provided along with research works performing these tasks. In Table 8, a list of downstream datasets utilized by different FMs is provided.

Another aspect to consider is how the performance evaluation is conducted. Among the surveyed articles, there are three different strategies that are employed.

1. *Zero shot evaluation:* In zero shot evaluation, the pre-trained model is directly used in a downstream task without probing or fine-tuning the pre-trained model with any samples of the downstream dataset. This provides a direct assessment of learned representation in the pre-training phase, i.e., evaluates the quality of the generated embeddings from the pre-trained model. This is the most common approach among the surveyed articles.
2. *Few shot evaluation:* In few shot evaluation, the pre-trained model sees only a few examples from the downstream task dataset. This is sometimes mentioned as K-shot evaluation where K is the number of data points seen by the model.
3. *Simple shot evaluation*: Simpleshot^164^ is a unique variation of the few shot evaluation methods which is only been explored in UNI. It combines few shot learning with a nearest-neighbor classifier.

Several factors are taken into account when choosing between the three evaluation strategies. The first is to assess how “task-agnostic” the FM is, i.e., how easily the FM can adapt to a variety of diagnostically challenging downstream tasks. The zero shot evaluation is the appropriate strategy in this case, as the FM was not trained directly for the purpose of solving a specific downstream task. The second factor is assessing the sample efficiency of the FM, i.e., how quickly the model learn/adapts if given a few examples.

Few-shot evaluation is the most intuitive strategy here, and by varying the number of examples in the few-shot setting, it is possible to assess the sample efficiency. Finally, the quality or robustness of the generated embeddings is another factor to consider. Simple shot evaluation is more appropriate here, as it can evaluate how well a model's pre-trained features cluster new task data.

Table 7 provides a comparison of the three evaluation strategies commonly used to assess FMs on downstream tasks, each offering distinct trade-offs across several criteria.

Zero-shot evaluation requires no labeled data and provides an unbiased representation assessment, making it ideal for fair cross-study comparison. It also offers low variance across runs, low risk of overfitting, and does not require any classifier training. Additionally, it is robust to example selection bias because no examples are selected. However, it does not allow model adaptation to the target task and is not resilient to domain gap, which can underestimate model potential. It also does not reflect real-world low-resource scenarios where some labeled data are typically available.

Few-shot evaluation allows model adaptation to the target task using a small number of labeled examples, making it resilient to domain gap and better at reflecting real-world low-resource scenarios. However, it is not robust to example selection bias, as results can vary depending on the chosen samples, leading to higher variance across runs. This also makes fair cross-study comparison more difficult. Furthermore, it requires classifier training and carries a higher risk of overfitting on small datasets.

Simple-shot evaluation offers a lightweight alternative that requires no classifier training and is robust to example selection bias. It provides low variance across runs, low risk of overfitting, and enables fair cross-study comparison. Like few-shot, it reflects real-world low-resource scenarios. However, it does not allow model adaptation to the target task, is not resilient to domain gap, and does not provide an unbiased representation assessment. Notably, it has not been widely explored in literature, having only been used in UNI,^31^ limiting conclusions about its general reliability. The choice of evaluation strategy should be guided by the goals of the study, and ideally, results should be reported across multiple strategies to provide a balanced assessment of model capabilities.

### Framework, benchmarking, and adaptation of FMs

There are several research works in CPath that do not directly propose a FM but introduce the framework of FMs, provide benchmarks and comparisons between FMs and adapt the FMs for efficient training. Because of so many existing research works in FM, there is no unified or consistent approach to accessing these models, making it difficult to replicate the results. Furthermore, due to the diversity of downstream tasks and datasets, it is inconvenient to alter each FM for each set of downstream tasks and datasets. Frameworks address this issue by providing a common interface and a consistent implementation method. Another drawback of having different FMs is that there is no agreed-upon way to compare them. Research works in benchmarking the FMs' approach to this issue by providing consistent comparison criteria and ranking them based on the performance metrics. FMs contain a vast number of parameters, making fine-tuning challenging; however, various adaptation strategies can help ease this process. Through strategies like parameter-efficient fine-tuning, prompting techniques, and in-context learning, it is possible to fine-tune FMs with minimal overhead.

#### Frameworks

eva^85^, ^165^ is a framework for VFMs in CPath which abstracts a lot of complexity of VFMs. In addition, it facilitates the reproducibility of VFMs for fair comparison and provides an interface to evaluate publicly available downstream datasets.

#### Benchmarking

Another category is benchmark analysis of FMs^78^, ^79^, ^81^, ^82^, ^84^, ^87^, ^89^ in CPath. The work in Lu et al.,^78^ benchmarks the performance of six FMs (CTransPath,^35^ PathoDuet,^23^ PLIP,^42^ CONCH,^41^ and UNI^31^) across five clinically relevant prediction tasks. The contribution of Mallya et al.,^79^ is benchmarking FMs, which include CTransPath,^35^ Lunit,^32^ Phikon,^33^ PLIP,^42^ UNI,^31^ and Virchow^24^ for ovarian cancer bevacizumab reatment response prediction. Another work^81^ analyzes the performance of FMs on a large and diverse dataset collected from two medical centers. The FMs (CTransPath,^35^ UNI,^31^ Virchow,^24^ and Prov-GigaPath^38^) were benchmarked on three broad downstream tasks, which include disease detection, biomarker prediction, and treatment outcome prediction. In Zheng et al.,^82^ the authors benchmarks PathCLP^40^ on various corrupted images (with seven corruption types) for two downstream datasets. The authors of Ref.^84^ investigate how layer selection for FMs (CTransPath,^35^ Phikon,^33^ Lunit,^32^ + other encoders) affects the downstream task performance. The work done in Alfasly et al.,^87^ analyzes different FMs and evaluates their performance on eight datasets. In Lai et al.,^89^ the authors evaluates the performance of histopathology-specific SSL methods for 17 unique tissue types and 12 unique cancer types.

#### Adaptation of FMs

Another category is the adaptation of existing FMs to carry out tasks such as low-resource fine-tuning^88^ and multi-modal prompt-tuning.^80^ The work carried out in Roth et al.,^88^ fine-tunes a FM with a single GPU and shows that it can outperform SOTA feature extractors. PathoTune^80^ adapts a visual- or pathology-specific FM to downstream tasks using multi-modal prompts. In Roth et al.,^83^ the authors proposes a task-specific visual prompting approach to tune VFMs. The work done in Ferber et al.^86^ utilizes in-context learning to learn from prompts without parameter updates. They specifically utilizes GPT-4 with vision capability and performs evaluation on three downstream datasets.

Other than the surveyed FMs, there are more recent works of FM in CPath like mSTAR.^166^ However, as it includes RNA-Seq data in addition to pathology reports it falls outside the scope of this article. Another FM we do not include is H-optimus-0^25^ developed by Bioptimus as it is not associated with a publication.

## Vision-language models

In this section, the recent works in CPath with VLMs are outlined. The details about architecture, used datasets and contribution of individual research work are listed in Table 9.

First, different categorizations of VLMs are provided (Section 4.1) to give insight into how VLMs are utilized to solve pathology-specific tasks. Second, the common architectural components in VLMs and adopted strategies are summarized (Section 4.2). Then, brief summary of models focusing on visual instruction tuning is provided (Section 4.3). Lastly, a brief overview is given for models that do not solve a direct pathology-specific task but perform other types of vision-language tasks (Section 4.4).

### Categorization of VLMs

A categorization of VLMs in CPath can be done based on the reason for using the language modality. Some research works solve tasks like caption generation^115^, ^150^, ^151^, ^152^, ^158^ or VQA,^51^, ^107^ which necessitates utilization of both vision and language modality because of the nature of tasks. For VQA or caption generation, the generated output from the model needs to be in language form, i.e., the model needs to perform a language task. On the other hand, other research works use language modality as a source of semantic information to be injected into information gained from vision modality. This additional semantic information can significantly boost the performance of the model which would not have been possible with a vision-only model. As an example, MI-Zero^71^ performs cancer subtyping, which is not a language task but utilizes pathology reports curated from the in-house dataset and TCGA as a source of semantic information.

Another categorization can be done by comparing the training approach with FMs. VLFMs are different from VLMs as traditional VLMs focus on solving one or two vision-language tasks like caption generation. However, as FMs become more prevalent recent works are starting to shift towards VLFMs. Recent works (PathGen-CLIP,^53^ Quilt-Net^54^) take FM-like approach to train their models and adapt them to different downstream tasks. On the other end of the spectrum, there are VLMs that solve only a single task. As shown in Fig. 17, VLMs and VLFMs should be viewed as a spectrum where if the number of tasks and pre-training data size increases it moves away from VLMs and towards VLFMs.

Owing to this, some VLMs follow the FM approach of pre-training the vision or text module. However, as pre-training from scratch requires a large amount of data and computing some VLMs use domain-specific vision modules from HIPT,^138^ CTransPath,^35^ IBMIL,^157^ etc. or language modules from PubMedBERT,^130^ BioClinicalBERT,^129^ etc. This allows the vision or language module to learn an initial vision or language representation space which might not be as rich as pre-training scratch but boosts the performance. A summary of the choice of encoder type and pre-training strategy is provided in Table 10.

Another categorization can be done by analyzing how language prior is incorporated into the model. Most works use text labels or captions curated from different sources, which provide the least amount of information. Recent works like FSWC,^111^ ViLA-MIL,^112^ and PEMP^149^ utilize prompting LLMs to increase the amount of semantic information that can be gained from text labels or captions. They prompt the LLMs to generate descriptions about a particular class label or of morphological or textural patterns about patches. On the other end of the spectrum is research work utilizing all information from pathology reports, which contain a lot of details. However, as shown in Fig. 18, the difficulty of curation and doing it at a scale to match the pathologist level also increases.

### Architectural components for VLMs in CPath

Even though there is a huge variety of vision and language modules used in VLMs, it is possible to identify common structures. In Fig. 19, the common architectural components used in different stages are outlined. In the pre-processing stage, LLMs like GPT-4, GPT-3.5, GPT-3.5-turbo, and GPT-3 are used to clean and refine captions or prompting them to generate descriptions. Examples of research work utilizing LLMs in the pre-processing phase include HistGen,^124^ FSWC,^149^ ViLA-MIL,^112^ FiVE,^119^ CPLIP,^123^ and PromptBio.^156^

Another key component of the architecture is the vision module or encoder, which converts the WSI patches to image embeddings. As shown in Fig. 19, it can be separated into three separate groups. The first group is vanilla CNNs that have ImageNet initialization or some kind of pre-training on pathology data. Note that most VLMs utilizing these architectures are not recent works (with the exception of Elbedwehy et al.^152^). Recent VLMs utilize ViTs, which is the second group containing several ViT variants. The third group is special encoders that are pathology domain-specific and optimized for encoding WSI patches. These are ideal for learning a rich representation without the cost of pre-training from scratch.

For language encoders, most works utilize BERT (PubMedBERT,^130^ BioClincialBERT^129^) or GPT (BioGPT,^66^ GPT 2^106^) variants and most are pre-trained on datasets from the biomedical domain. Pre-training on biomedical and clinical corpora ensures their outputs reflect medically accurate and contextually appropriate language.

Another type of language module is the caption/text generation module which rather than encoding text into embedding, generates text sequences. Earlier works included RNN, LSTM, and Transformer decoders and recent works include LLMs like BioGPT 2,^66^ BioClinicalBERT,^129^ Flan-T5,^114^ PaLM-2,^137^ etc. The generation of text sequence process works by predicting the next token based on the previous sequence of tokens. Additionally, as the module is a part of VLM, attention to the visual features is used as guidance.

The last kind of architectural components are modules that align the generated visual and text features or fuse the features to get a combined representation. The most common vision-language alignment or fusion module is the CLIP model and its variations. However, some works come up with custom approaches to align vision and language representations. One such approach is using cross-attention layers, which allow the text tokens to attend to the visual tokens, learning rich inter-modal interactions. This performs vision-language alignment by focusing on semantically meaningful visual cues.

### Visual instruction tuning in CPath

Vision instruction tuning refers to the process of adapting a VLM to follow specific, structured visual instructions by training it on datasets that pair images with corresponding natural language prompts and responses. The goal is to teach the model to interpret and respond to multi-modal instructions effectively, enabling it to perform complex tasks based on visual cues. There are four research works/models that fall in this category (PA-LLaVA,^167^ PathInsight,^168^ CLOVER,^169^ PathGen-LLaVA,^53^ and Quilt-LLaVA^58^).

In PA-LLaVA,^167^ a domain-specific large language-vision assistant is proposed which is a LLaVA-based^55^ model. In terms of architecture, PLIP^42^ is used as the vision encoder and LLama3^170^ is used as the LLM (in conjunction with LoRA^122^). A connector module that consists of self-attention and cross-attention blocks is leveraged to project image embeddings to match the dimension of language tokens. PathInsight^168^ leverages three different multi-modal models LLaVA,^55^ Qwen-VL,^171^ and InternLM^172^ (in conjunction with LoRA^122^ or full-parameter tuning). As the vision encoder, either ViT or CLIP is used. CLOVER^169^ proposes a framework for cost-effective instruction learning in CPath. It utilizes Quilt-1M as the vision-language dataset, BLIP-2^136^ for vision-language pre-training and a frozen visual encoder (EVA-ViT-G/14^173^) and a frozen LLM (FlanT5^114^ or Vicuna^56^). The next work PathGen-LLaVA^53^ replaces the vision encoder for the CLIP model in LLaVA-v1.5^77^ with PathGen-CLIP^53^ and as the frozen LLM Vicuna^56^ is used. The last work in this category Quilt-LLaVA^58^ is initialized with the general-domain LLaVA. Then, it is tuned with QUILT for histopathological domain-alignment and finally, Quilt-instruct is used for visual instruction tuning.

### Related works for VLMs in CPath

There are several research works that do not directly utilize VLMs to solve a task in the pathology domain, but investigate or use VLMs for other reasons. One example is Thota et al.,^174^ which investigates the effect of projected gradient descent adversarial perturbation attack^175^ on PLIP^42^ architecture. Another work by Lucassen et al.^176^ provides a pathology report processing workflow that can be used for VLMs in CPath.

Another category of VLMs in CPath that does not directly solve pathology-specific tasks is text-guided diffusion models for image generation. However, these models can perform tasks like virtual stain transfer which can later be used to solve a pathology-specific task. Another use case is extending dataset size with generated synthetic images. One such work is PathLDM,^177^ that performs text-to-image generation on the TCGA-BRCA dataset.

## Conclusion

In recent years, the number of research works in CPath with FMs and VLMs has increased significantly which provides an indication about how CPath will evolve in the next couple of years. FMs accelerated the development of targeted models for clinical applications by providing robust, transferable representations and reducing the reliance on large annotated datasets. With minimal fine-tuning, they can be adapted to a wide range of challenging diagnostic tasks, reducing the need for extensive training. Many of these works put significant computing resources into training FMs on massive pre-training datasets or coming up with novel strategies to push more language prior knowledge into VLMs. Whereas VLMs are not yet routinely integrated into clinical practice, they are likely to be adopted when rigorous clinical validation studies become available. In the near future, the VLFMs, which bring together the benefits of both FMs and VLMs, are going to be the dominant model with the prospect of being employed in the real world.

Several emerging directions are expected to further shape this field. Current VLMs primarily combine vision and language, but future models are likely to move towards true multi-modal integration by jointly analyzing histopathology images with genomic profiles, radiology scans, electronic health records, and molecular assays for a more holistic understanding of the patient's condition. Translating these models into clinical practice remains a key challenge, requiring regulatory approval, integration with existing lab systems, and consistent performance across diverse patient populations. Rather than replacing pathologists, FMs and VLMs are well positioned to serve as diagnostic partners by generating interpretable outputs, flagging ROIs, and providing differential diagnoses, thereby reducing diagnostic workload and especially in resource-limited settings.

The emergence of spatial transcriptomics and spatial proteomics also presents new opportunities, enabling FMs to link tissue morphology with spatially resolved molecular data to better understand the tumor microenvironment and tissue heterogeneity. Furthermore, FMs and VLMs hold significant potential in precision oncology, where they can assist in predicting treatment response and identifying actionable biomarkers directly from histopathology slides.

Realizing this potential will require continued collaboration between computational researchers, pathologists, and clinicians. This review provides a comprehensive overview of the current landscape of FMs and VLMs in CPath, which will aid future researchers in navigating this rapidly evolving field.

## Declaration of competing interest

The authors declare the following financial interests/personal relationships which may be considered as potential competing interests: The editor is also an author of this manuscript; however, they had no involvement in editorial decision-making or peer-review process for this manuscript.
